# Effects of Cardiovascular Disease Risk Factors, Musculoskeletal Health, and Physical Fitness on Occupational Performance in Firefighters—A Systematic Review and Meta-Analysis

**DOI:** 10.3390/ijerph191911946

**Published:** 2022-09-21

**Authors:** Jaron Ras, Andre P. Kengne, Denise L. Smith, Elpidoforos S. Soteriades, Rucia V. November, Lloyd Leach

**Affiliations:** 1 Department of Sport, Recreation and Exercise Science, Faculty of Community and Health Sciences, University of the Western Cape, Cape Town 7535, South Africa; 2 Non-Communicable Diseases Research Unit, South African Medical Research Council, Cape Town 7505, South Africa; 3 Health and Human Physiological Sciences, Skidmore College, Saratoga Springs, New York, NY 12866, USA; 4 Healthcare Management Program, School of Economics and Management, Open University of Cyprus, Nicosia 2220, Cyprus

**Keywords:** firefighters, cardiovascular health, risk factors, musculoskeletal health, physical fitness, occupational performance

## Abstract

**Background**: Firefighting is a strenuous occupation, which necessitates that firefighters stay in good physical condition and maintain adequate cardiovascular and musculoskeletal fitness to perform their duties with minimal health and safety risks. The aim of this review is to determine the effects of cardiovascular disease risk factors, musculoskeletal health, and physical fitness on the occupational performance of firefighters. **Methods:** PubMed/Medline, SCOPUS, Web of Science, EBSCOHost, and ScienceDirect were searched without time-restriction. The appraisal tool for cross-sectional studies and the Critical Appraisal Skills Programme toolkit were used to conduct the methodological assessment. Data were analyzed using Review Manager 5.3, and MedCalc^®^ statistical software. **Results:** Age had a moderate effect on occupational performance (Z = 5.15, *p* < 0.001), whereas gender had a large effect size on occupational performance (Z = 4.24, *p* < 0.001). A significant moderate negative correlation was found between cardiorespiratory fitness and occupational performance (R = −0.584, *p* < 0.001). Significant low negative correlations were found between upper body endurance (R = −0.344, *p* < 0.001), abdominal endurance (R = −0.308, *p* < 0.001), grip strength (R = −0.421, *p* < 0.001), upper body strength (R = −0.318, *p* < 0.001), and lower body strength (R = −0.216, *p* = 0.020) and occupational performance. **Conclusions:** Aged firefighters with poor body composition and lower levels of physical fitness performed worse on all occupational performance tasks.

## 1. Introduction

Firefighting is a hazardous occupation that places high physiological and psychological stressors on firefighters, thereby, posing significant risks to their health and wellbeing [1,2,3]. In addition, the environmental stressors include extreme temperatures, and hazardous chemicals and fumes [3,4,5,6,7,8]. The extreme environmental conditions necessitate that firefighters wear heavy, insulated personal protective equipment (PPE), which often includes self-contained breathing apparatus (SCBA) that places tremendous strain on their cardiovascular system [6,8,9]. Moreover, firefighters are required to perform strenuous work duties, such as emergency rescues, first aid and resuscitation, and emergency extrication from vehicles, all while working irregular hours [1,8,10,11]. These types of strenuous and irregular working conditions place significant strain on the musculoskeletal and cardiovascular systems of firefighters, increasing the risk of serious injuries and sudden cardiac events, while on duty [1,12,13,14]. 

Existing research indicates that many firefighters have multiple cardiovascular disease (CVD) risks factors or poor overall cardiovascular health [3,15,16,17,18,19], poor musculoskeletal health [20,21,22,23] and inadequate physical fitness [24,25,26,27], which significantly and negatively affect their occupational performance [20,21,28,29,30,31]. Extensive scientific literature indicates that among emergency services professionals, firefighters have one of the highest percentages of mortality (45%) due to sudden cardiac death (SCD), with the majority related to underlying CVD risk factors [1,10]. These deleterious consequences are likely, at least partially, attributed to inadequate physical fitness, which invariably results in overexertion and increased cardiovascular strain [7,8,32], particularly when wearing full protective gear. Under these conditions, studies have shown the induction of maximum physiological responses, and often with adverse health outcomes [9,32,33]. In addition, firefighters have been reported to have the highest incidence of musculoskeletal injuries among all emergency services personnel [1], which is likely attributable to a combination of the weight of the PPE [32], the high prevalence of obesity [34,35,36], the necessity for sudden changes in posture and gait on rescue [35,36] and the high musculoskeletal demand of their professional duties [37,38,39]. The combination of extraordinary musculoskeletal health demands, deteriorating cardiovascular health and inadequate physical fitness in many firefighters, may lead to significant morbidity and mortality in this population [40,41]. In addition, the progressively deteriorating cardiovascular and musculoskeletal health with increasing age, and the overall poor physical fitness significantly and negatively affect firefighters’ occupational performance [15,17,20,21,28,29,30]. Consequently, firefighters who are unable to perform their duties with sufficient competency and rigour are at risk of underperformance while on-duty [30,42], thereby, placing their lives as well as those of other civilians at increased risk, and increasing the potential loss of property and infrastructure. Firefighters who are not fit for active duty may be at increased risk of sustaining cardiovascular events and musculoskeletal injuries [27,30,43,44]. 

Measuring firefighters job performance while on active duty is an inherently difficult and costly task, due to the physical nature of their occupation and the high likelihood of equipment becoming lost or damaged [45]. This is particularly true for fire departments in developing countries or those fire departments that cannot afford to equip firefighters with this equipment [46,47]. Therefore, to assess firefighters’ work ability, fire and rescue departments use occupational simulation protocols to determine if firefighters are able to perform their duties with sufficient rigor [6,28,33]. Previous research has indicated that occupational simulation protocols are the closest representation of the stressors of firefighting [48]. Globally, an alarming number of firefighters are at increased cardiovascular disease (CVD) risk, while suffering from multiple musculoskeletal disorders and operating under suboptimal levels of physical fitness. This negatively effects their occupational performance and limits their ability to cope with the on-duty demands [2,6,31,39,49,50]. However, there have been no previous systematic reviews investigating the effects of CVD risk factors, musculoskeletal health, and physical fitness on the occupational performance of firefighters, which motivated the need for the present study. 

The relative lack of systematic reviews on this current topic was somewhat surprising, given the nature of the occupation. Providing more information on the effect that cardiovascular disease risk factors, musculoskeletal health and physical fitness have on occupational performance may provide valuable evidence in informing policy makers and fire departments. For more information on the aim and objectives of this review, please refer to the published protocol: Ras et al. [51]. Briefly, the aim of this systematic review and meta-analysis was to determine the effects of CVD risk factors, musculoskeletal health and physical fitness on the occupational performance of firefighters. The objectives of the review were (i) to investigate the effects of cardiovascular health on the occupational performance of firefighters; (ii) to investigate the effects of musculoskeletal health on the occupational performance of firefighters; (iii) to investigate the effects of physical fitness on the occupational performance of firefighters, (iv) and, to investigate the relationship between cardiovascular health, musculoskeletal health and physical fitness on the occupational performance of firefighters. 

## 2. Materials and Methods

**Figure 1:**
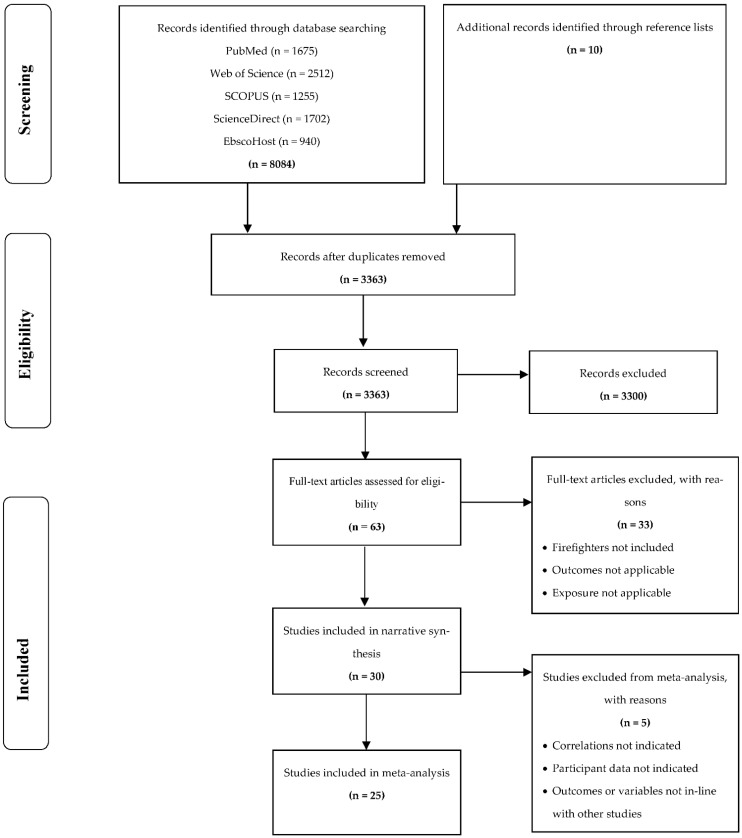
Flow chart of study selection in narrative review: Adapted from Moher et al. [54].

The guidelines for Meta-analysis of Observational Studies in Epidemiology (MOOSE) and Quality of Reporting of Meta-analysis (QUOROM) guided our methods when conducting this review [52,53]. When considering studies for this review, the PRISMA guidelines for systematic reviews was followed, and the outcomes for each step was described in a flow-diagram [54] (Figure 1). 

### 2.1. Summary of Methods 

The study design of choice is a quantitative systematic review, where participants included adult, full-time, part-time and volunteer firefighters between the ages of 18 to 65 years. The exposures assessed included cardiovascular health, musculoskeletal health, and physical fitness in relation to the occupational performance of firefighters. The inclusion criteria were as follows: (i) studies that recruit full-time adult firefighters, with no limitations to publication year; (ii) studies investigating the effects of cardiovascular health, musculoskeletal health and/or physical fitness on the occupational performance of firefighters; (iii) studies available in full-text. Exclusion criteria included: (i) studies focusing on other outcome measures as the main exposures or outcomes; (ii) systematic reviews or other types of reviews; (iii) articles that are non-English. The protocol for this study has been published and more information on the methods involved in the current manuscript may be found at: Ras et al. [51].

### 2.2. Search Strategy for Identification of Studies

A detailed literature search was conducted by the two primary reviewers (JR and RN), tasked with independently identifying studies, extracting the data, verifying the data collected and grading the quality of the results. JR was the principal investigator tasked with data analysis, narratively synthesising the data and writing up of the systematic review. A third reviewer (LL) was tasked with adjudicating and resolving any disagreement between the two independent reviewers. 

### 2.3. Electronic Literature Search

The following journal databases were searched: PubMed/Medline, SCOPUS, Web of Science, EBSCOHost and ScienceDirect with no limitation to publication year. Keywords and medical subject heading (MeSH) terms were used in various arrangements depending on the specific database. A combination of the appropriate terms (search string) was used to ensure the inclusion of the relevant components of the participants, exposure, comparison, and outcome (PECO). The details of the search strategy can be found in Supplementary S1.

### 2.4. Additional Searches for Grey Literature

The search strategy was completed by searching the following databases for grey literature: Google, Google Scholar and Networked Digital Library of Theses and Dissertation. 

### 2.5. Selection of Studies

All studies, as full-text articles, that met the inclusion criteria were selected for screening. Every attempt was made to contact the authors for full-text articles or missing data. Thereafter, the full-text articles were assessed independently by two reviewers using the Rayyan^®^ intelligent systematic review (RIS) tool [55]. When screening the studies, three categories were used, namely, included, excluded and unsure. Any uncertainties regarding study inclusion were discussed between the two reviewers. In the event of disagreement, a discussion was held with the third reviewer, and resolved by the latter. 

### 2.6. Data Extraction and Data Management

A researcher-generated data extraction form was used (Supplementary S2 and Supplementary S3). The information extracted was the general study details, such as authors, date of study publication, study title, study design and country of study, the exposure assessed, and the outcome measures. Study characteristics were collected, such as sampling method and sample size, and details of the participants. In addition, the details of exposure and the outcome variables were extracted, i.e., the study must have reported on at least one of the exposure variables in relation to firefighter occupational performance.

#### 2.6.1. Critical Appraisal of Included Studies

The appraisal tool for cross-sectional studies (AXIS checklist) (Table 1) [56] and The Critical Appraisal Skills Programme (CASP) toolkit (Middle Way, Oxford, UK) (Table 2) (https://casp-uk.net/casp-tools-checklists/ (accessed on 1 March 2021)) were used to conduct the methodological assessment of each study included. The CASP toolkit (Middle Way, Oxford, UK) was previously used in systematic reviews on firefighters and tactical personnel to assess study methodologies, and allows for fair and equitable assessment of a variety of study types. The AXIS toolkit was shown to be a reliable and valid tool for assessing the quality of cross-sectional studies [56]. 

#### 2.6.2. Classification of Age and Obesity and Physical Fitness for Meta-Analysis

Age was classified as male firefighters over the age of 45 years, and obesity was classified as a BMI of 30 kg·m^2^ or higher or a bodyfat percentage (BF%) over 25%. For cardiorespiratory fitness, only studies that included either absolute (mL·kg·min) or relative (L·min) $\dot{V}$O2max were used. These estimates included both from direct gas analysis and those estimated with maximal or submaximal $\dot{V}$O2max. For upper body and abdominal muscular endurance, the push up and sit ups endurance tests were preferred. For upper body strength grip strength and the bench press were used as the preferred measures, and for lower body only studies including the leg press or squat were included. For flexibility, the sit and reach test was preferred. These physical fitness tests were favored due to their frequency of use across multiple studies when used to indicate overall health-related physical fitness in firefighters.

### 2.7. Data Analysis

#### 2.7.1. Assessment of Overall Effect Size

The outcome measure (occupational performance) was analysed as a continuous variable. The mean difference (MD) and standardized mean difference (SMD), with 95% confidence intervals (CI), of estimation was used to estimate the effect, using the inverse variance method of meta-analysis, between cardiovascular health and occupational performance and physical fitness and occupation performance in firefighters [81]. For the correlation analysis, MedCalc^®^ statistical software Ltd., Ostend, Belgium (version 20.104), was used to perform the correlation meta-analysis. Investigators grouped the “R” values according to cardiovascular disease risk factors, physical fitness components and overall performance, and combined them into a single representative effect estimate [82]. Meta-analysis techniques were applied using the number of studies, original “R” values and sample sizes to generate the pooled “R” values between each cardiovascular health component, fitness component and job task component [82]. Where one study, or insufficient studies were present, a meta-analysis on the pooled “R” was not calculated [82]. The original “R” values were converted to a common test metric using the Fisher’s “R” to “Z” transformation [82]:

$$Z_{\mathrm{ri}}=\frac{1}{2}\ln\left(\frac{1+ri}{1-ri}\right)\;s\frac{2}{z}=\frac{1}{n-3}$$

The Fisher’s Z values from the original studies were combined using random effect models for all analysis’ performed [82]. The following was used to indicate the strength of correlation, 0.00 to 0.30 (−0.00 to −0.30) for negligible correlation; 0.30 to 0.50 (−0.30 to −0.50) for low correlation; 0.50 to 0.70 (−0.50 to −0.70) for moderate correlation; 0.70 to 0.90 (−0.70 to −0.90) for high correlation and 0.90 to 1.00 (−0.90 to −1.00) for very high correlation [82]. 

#### 2.7.2. Assessment of Heterogeneity

Heterogeneity was evaluated using the Chi-square test, I^2^ test and Cohen’s Q test [83]. The following was used to explain I^2^ statistics: (1) 0% to 30%: may not be important; (2) 31% to 60%: may indicate moderate heterogeneity; (3) 61% to 80%: may indicate substantial heterogeneity; (4) 81% to 100%: considerable heterogeneity. Regardless of whether homogeneity or heterogeneity were present between studies, a random-effects model was preferred in order to maintain consistency in the interpretation of results [83]. To assess the risk of bias between studies, the Egger’s test and Begg’s test were performed. 

#### 2.7.3. Subgroup Analysis and Investigation of Heterogeneity

When heterogeneity was present, a subgroup analysis was performed to explore the sources of heterogeneity [81,83]. Where applicable, subgroup analysis included the following: weight of personal protective equipment (PPE), the sex of the firefighters (male and females), the number of tasks performed and if tasks were sequentially or discretely conducted, for full-time firefighters. For the weight of PPE, studies that had a combined weight of PPE above 22 kgs. The number of tasks performed included studies where firefighters performed five or more tasks during the occupational simulation protocols. Sequential tasks included studies that included tasks that were performed sequentially, i.e., followed a specific order, whereas discrete tasks included studies that had no specific order. An additional subgroup analysis was included for cardiorespiratory fitness, which included studies that estimated cardiorespiratory fitness directly by using gas analysis. Although all exposures were measured using a standard physical ability test or simulated work-related tasks, the methods used could be different, which required comparing and converting certain measurements to produce similar findings for comparison. 

## 3. Results

### 3.1. Study Selection

Initially, the electronic database searches yielded 8084 publications, with an addition of 10 studies found through reference list searching (Figure 1). After removal of duplicates, 3363 studies remained and were screened using title and abstract information. Of these, 3300 studies were excluded for not meeting the inclusion criteria, leaving 63 studies that were designated for full-text screening. A total of 33 studies were excluded after screening the full text, and 30 studies were eligible to proceed to data extraction. After data extraction, 25 studies were included for the final meta-analysis.

### 3.2. Assessment of the Strengths and Weaknesses of Studies

The strengths and weaknesses of the studies were assessed and the most frequent weakness of the studies, according to the AXIS checklist, were: (a) the sample size was not justified (13/27); (b) the sample frame not taken from an appropriate sample base (22/27); (c) the sample selection not likely to select participants that represented the target population (21/27) (Table 1). These weaknesses were largely due to the nature of the study types and the relatively small sample sizes. The quality of the included studies was acceptable. A score of 15 point was given a score of “moderate”, 16 to 17 point given a score of “good” and scores between 18−19 given a score of “high” quality (Table 1). Scores of lower quality (<15) were excluded from this review. Then strengths and weaknesses of cohort studies were assessed according to the CASP checklist for cohort studies, and all studies were of high quality, with N/A given to two questions, namely: (a) What are the results of this study? and (b) How precise are the results? (Table 2).

### 3.3. Study Characteristics

The included studies encompassed 27 cross-sectional studies and three cohort studies conducted between the period of 1987 and 2022, and included 2585 firefighters. Studies were conducted in different global regions and encompassed multiple variations of occupational simulated tasks. A summary of the included studies is presented in Table 3.

### 3.4. Cardiovascular Disease Risk Factors, Musculoskeletal Health and Occupational Performance 

The results indicated that only two cardiovascular disease risk factors were consistently studied according to occupational performance in firefighters, and included age and obesity (Table 3). The studies reported that older firefighters’ completion times and performance on each individual task was significantly lower compared to younger firefighters [39,58,74,77]. When firefighters were aged (over 45 years in males), overall performance was significantly reduced. Obesity was reported to significantly reduce overall occupational performance and performance on each individual task [30,57,60,65,73,74,77,79,80]. Resting diastolic blood pressure and diastolic blood pressure at completion of the simulation event was significantly related to occupational performance in firefighters [72,84]. The results indicated that, although males tended to be at higher risk for cardiovascular disease, they also performed significantly better overall and in each occupational task compared to female firefighters [39,58,64,72]. Only one study was found that investigated the relationship between musculoskeletal health and occupational performance in firefighters. Although not statistically significant, the study found that firefighters who reported having moderate-to-severe muscle and joint problems took approximately 10 s longer to complete the five flights of stairs while carrying a 22 kg high-rise pack than firefighters not reporting those problems [29].

### 3.5. Physical Fitness and Occupational Performance in Firefighters

The results indicated that cardiorespiratory fitness [28,30,58,60,61,64,65,66,68,70,80,84], muscular endurance [30,42,57,62,65,66,70,73,77,80,84] and muscular strength [30,42,57,62,72,73,77,80,84] were significantly related to overall occupational performance in firefighters (Table 3). In addition, cardiorespiratory fitness was significantly related to the time required to complete the stair climb [30,31,42,60,62,65], hose drag [30,31,42,66], crawl [62,66], ladder raise [62,66], terrain crossing [62], demolition [62], rolled hose lift and move [57], equipment carry [66], hose pull [62], victim rescue [30,42,60,62,65,66], forcible entry [66] equipment hoist [42], and saw hold/cutting [30,61]. Upper body endurance was significantly related to tasks requiring upper body work, such as the hose drag [30,57,66,77], hose pull [61], hose connect, victim rescue [30,57,66,77], hose pull [61], rolled hose lift and move [57], crawl [66], ladder raise [66] terrain crossing [62], demolition [62], equipment carry [66], saw hold, forcible entry [57,66,77] and equipment hoist, and also a lower extremity dominated task such as the stair climb [30,57,77]. Abdominal endurance was significantly related to the stair climb [30,42,57,61,77], hose drag [30,42,57,66,77], hose pull [61], hose connect, victim rescue [30,42,57,61,66,77], hose pull [61], rolled hose lift and move [57], crawl [66], ladder raise [66], terrain crossing [62], demolition [62], equipment carry [66], forcible entry [57,66,77] and equipment hoist [42,77]. Grip strength was significantly related to hose drag [30,31,42,57,77], victim rescue [30,42,57,61,77], rolled hose lift and move [57], crawl, hose pull [61], terrain crossing [61], demolition [61], forcible entry [57,77] and equipment hoist [42,77], however, grip strength consistently appeared to have a stronger relationship with overall performance and each specific occupational task. Upper body strength was significantly related to hose drag [30,42,57], victim rescue [30,42,57] and rolled hose lift and move [57]. Surprisingly, lower body strength was most consistently reported not to be significantly related to stair climb times [30,31,42,57,76] in firefighters. However, lower body strength was significantly related to the hose drag [30,31,42,57], hose pull, victim rescue [30,42,57], and rolled hose lift and move [57]. Flexibility has been reported to be significantly related to stair climb times [77], however, in one study no relationship was found between these variables. A study reported a relationship between quadriceps muscle diameter and stair-climb time (R = 0.560, *p* < 0.001) [67], however, this does not coincide with the results of previous literature.

**Table 3:** Study characteristics of included studies.

References	Study Design, Setting and Sample	Sample	Participant Information and Physical Fitness Measures	Occupational Performance Measures	Testing Procedure Details	Outcome
Chizewski et al. [66]	Cross-sectional study USA, Midwestern states	89 male firefighter recruits	Age: 26.8 ± 4.2 years Body mass: 89.24 ± 16.33 kgs Height: 1.78 ± 0.07 m BMI: 28.11 ± 4.19 kg·m^−2^ 1.5 Mile Run, push-ups, sit-ups bench press, flexibility, vertical jump.	Kiser Sled SCBA Crawl Victim Drag Hose Advance Equipment Carry Ladder Raise	Full PPE SCBA gear Sequential tasks 20.4 kgs	Significant relationships between cardiovascular endurance (r = −0.49, *p* < 0.01), bench press (r = −0.51, *p* < 0.01), push-ups (r = −0.38, *p* = 0.01), sit-ups (r = −0.41, *p* < 0.01), power (r = −0.32, *p* < 0.01) and total firefighting ability (total completion time).
Davis et al. [74]	Cross sectional study USA, Washington D.C	100 full-time male firefighters	Age: 33.1 ± 7.63 Height: 176.7 ± 5.43 cm Weight: 83.4 ± 10.94 kgs LBM: 65.8 ± 5.98 kgs BF%: 21.1 ± 6.69% $\dot{v}$O2max: 39.60 ± 5.94 mL·kg·min. Treadmill test, handgrip strength, sit-ups, push-ups, sit-and-reach.	Ladder extension Standpipe carry Hose pull Simulated rescue Simulated forcible entry	Full PPE SCBA gear Sequential tasks 24 kgs	Significant predictors of performance on simulated tasks included the firefighters’ lean body mass, maximal heart rate, cardiorespiratory fitness, age, and BF%. High muscular strength and endurance and near maximal aerobic capacity was necessary to complete simulated tasks.
Elsner and Kolkhorst [68]	Cross-sectional study USA, San Diego	20 male firefighters	Age: 37.4 ± 8.5 years Height: 178 ± 6 cm Weight: 86.8 ± 8.9 kgs Body fat: 16.9 ± 4.7% Time: 11.65 ± 2.21 min Average $\dot{v}$O2: 29.1 ± 8.0 mL·kg·min Treadmill test	Hose advance and connect ladder carry and extension Donning their SCBA Advancing two sections of a fire hose Breach Stair climb Equipment hoist. Hose advance. stair decent Search and rescue	Full PPE and SCBA gear Sequential tasks	There was a moderately strong inverse relationship between $\dot{v}$O2max and performance time as well as a strong positive relationship between $\dot{v}$O2max and average $\dot{v}$O2 during the firefighting protocol.
Henderson et al. [80]	Cohort study USA, Milwaukee	306 male and female firefighter recruits	Age: 26.1 ± 4.7 years Height: 180.5 ± 6.4 cm LBM: 74.4 ± 8.1 kgs BF%: 13.3 ± 4.5% Step test, bench press, lat pull-down, grip strength, sit-ups	Stair climb Hose hoist Forcible entry Hose advance Victim rescue	Full PPE and SCBA gear Sequential tasks 29.3 kgs	BF% (r = −0.17) and age (r = −0.03) was negatively correlated with combat test performance. Absolute $\dot{v}$O2max (r = 0.43), bench press (r = 0.33), grip strength (r = 0.50), sit-ups (r = 0.31) were positively correlated with combat test performance.
Kleinberg et al. [67]	Cross-sectional study USA, North Carolina	46 male firefighters	Age: 37.6 ± 7.2 years Stature: 180.2 ± 6.9 cm Body mass (kgs) 108.0 ± 19.8 kgs BMI: 33.1 ± 4.7 kg·m^−2^ Quadriceps cross-sectional area (Q_CSA_) (cm^2^/kgs): 0.50 ± 0.07 Quadriceps echo intensity (Q_EI_): 109.3 ± 13.9	Stair-climb (s)	Fitted with weighted vest to simulate weight of PPE 22.7 kgs	Quadriceps cross-sectional area (QCSA) and quadriceps echo intensity (QEI) were significantly associated with stair-climb time (r = 20.492, *p* = 0.001; r = 0.363, *p* = 0.013, respectively). QCSA and QEI as significant predictors of stair-climb time (r = 0.560, *p* < 0.001) and a VIF of 1.046.
Lindberg et al. [62]	Cross sectional study Northern Sweden	38 male and female full-time, volunteer firefighters and civilians.	Age: 34 ± 9.8 years Weight: 78 ± 11.1 kgs Height: 177.2 ± 7.9 cm BMI: 25 ± 2.7 kg·m^−2^ Grip strength, sit-ups, grip endurance, squat endurance, bench press endurance, chin ups, dips, upright barbell row, standing broad jump, barbell shoulder press	cutting Stairs Hose pulling Demolition Victim rescue Terrain crossing	19 kgs	Significant correlations were present between all field tests and all the firefighter specific tasks (r = 0.45 to 0.85).
Lindberg et al. [61]	Cross sectional study Northern Sweden	38 male and female full-time, volunteer firefighters and civilians.	Age: 34 ± 9.8 years Weight: 78 ± 11.1 kgs Height: 177.2 ± 7.9 cm BMI: 25 ± 2.7 kg·m^−2^ Treadmill test, track running, step test, rowing.	cutting Stairs Hose pulling Demolition Victim rescue Terrain crossing	24 kgs	Absolute and relative $\dot{v}$O2max were significantly correlated to cutting (r = 0.55; r = 0.47), stairs (r = −0.75; r = −0.52), pulling (r = −0.74; r = −0.46), demolition (r = −0.79; r = −0.57), rescue (r = −0.79; r = −0.48) and terrain (r = −0.70; r = −0.74) performance.
MacDermid et al. [29]	Cross-sectional study Canada, Hamilton	293 male and female firefighters	Age: 42.6 ± 9.7 years Height: N/A Weight: N/A BMI: N/A	Work Limitations Questionnaire (WLQ-26) Hose Drag Stair Climb with a High-Rise Pack	Not specified Discrete tasks Not specified	Firefighters who reported having moderate to severe muscle and joint problems took approximately 10s longer to complete the stair climb task than did firefighters not reporting those problems.
Michaelides et al. [73]	Cross-sectional study USA, Arkansas	38 experienced volunteer firefighters	Age: 32.25 ± 6.07 years Weight: 96.1 ± 16.4 kgs Height: 178.21 ± 7.35 cm BF%: 21.78 ± 6.22% Abdominal strength, Relative power (vertical jump), Power (vertical jump), grip strength, bench press, squat, Sit and reach, Relative power (step test), Power (step test), Push-ups, Sit-ups	Stair climb, Rolled hose lift, and move, Keiser sled, Hose pull and hydrant hookup, Rescue mannequin drag, Charged hose advance	Full PPE and SCBA gear Sequential tasks 22.68 kgs	Upper body muscular endurance (push-ups to exhaustion) and upper body strength (1-RM bench press) were significantly inversely related with the total completion time the test (AT score; *p* < 0.01). In addition, there were significant positive associations (*p* < 0.01) between %BF and RHR variables and time to complete the AT. Flexibility, t(36) = 2.71, *p* < 0.05, %BF, t(36) = 3.11, *p* < 0.05, 1-RM bench press, t(36) = −2.24, *p* < 0.05, and 1-RM squat, t(36) = −2.06, *p* < 0.05, fitness parameters contributed significantly to the predictive power of firefighters’ AT performance.
Michaelides et al. [57]	Cross-sectional study USA, Arkansas	90 full-time male firefighters	Age: 32.25 ± 6.07 years Height: 181.16 ± 6.62 cm Body weight: 97.04 ± 15.51 kgs Age: 33 ± 67years Body fat: 23.05 ± 5.58% BMI: 29.55 ± 3.67 kg·m^−2^ Waist circumference: 97.33 ± 10.96 cm Abdominal strength, Relative power (vertical jump), Power (vertical jump), grip strength, bench press, squat, Sit and reach, Relative power (step test), Power (step test), Push-ups, Sit-ups	Stair climb Rolled hose lift, and move Keiser sled Hose pull and hydrant hookup Rescue mannequin drag Charged hose advance	Full PPE and SCBA gear Sequential tasks 22.68 kgs	Negative correlations indicated that higher performance on the fitness variables were associated with faster completion of the AT test, thus higher firefighting performance. Poor performance on the AT was significantly correlated (positive correlations) with high resting heart rate, body mass index (BMI), BF%, age, and waist size. Results showed that abdominal strength (t [53] = 22.94, *p* = 0.01); power, step test (t [53] = 22.37, *p* = 0.05); push-ups (t [53] = 1.97, *p* = 0.05); resting Hr (t [53] = 2.64, *p* = 0.05); and BF% (t [53] = 4.29, *p* = 0.01) contributed significantly to the predictive power of firefighters’ AT performance
Misner et al. [76]	Cross-sectional study USA, Chicago	150 female firefighter applicants	Age: 27.1 ± 4.5 years Height: 164.9 ± 5.6 cm Body mass: 63.4 ± 7.9 kgs BF%: 19.0 ± 5.9% LBM: 50.8 ± 4.3 kgs Leg press, bicycle ergometer, vertical jump, Wingate anaerobic test.	Stair climb test	Harness containing air pack 13.1 kgs	Stair climb performance was significantly correlated with age, lean body mass, vertical jump and peak power
Myhre et al. [65]	Cross-sectional study USA	222 male and female firefighters	Age: 30.4 ± 9.3 years Height: 178.6 ± 7.6 cm Weight: 83.5 ± 13.1 kgs BF%: 20.1 ± 6.9% Cycle ergometer test, bench press, upright forearm curl, upright row, barbell raise and lower.	“crash” aircrew rescue Search and rescue	Full PPE and SCBA gear Sequential tasks 22.2 kgs	Recue time was positively correlated to age (r = 0.38), and BF% (r = 0.36) and negatively correlated to $\dot{v}$O2max (r = −0.36), bench press (r = −0.18) and abdominal curl (r = −0.25).
Nazari et al. [31]	Cross-sectional study Canada, Ontario	46 males and 3 females firefighters	Age: 33.66 ± 9.19 years Height: 1.81 ± 0.08 cm Weight: 90.35 ± 13.22 kgs BMI: 27.53 ± 3.56 kg·m^−2^ $\dot{v}$O2max: 40.30 ± 6.25 mL·kg·min Cardiorespiratory fitness NIOSH lower limb strength combined grip strength	Stair climb, Hose drag	Full PPE and SCBA gear Discrete tasks 22.7 kgs	A negative correlation was present and indicated that higher $\dot{v}$O2max and/or strength levels were associated with faster completion of tasks Grip strength (r = −0.30) and CRF (r = −0.25) was negatively correlated to hose drag task. CRF was negatively correlated to the stair climb (r = −0.31). In predicting hose drag completion times, firefighters’ age and right grip strength scores were shown to be the most statistically significant.
Perroni et al. [85]	Cross-sectional study Italy, Rome	20 full-time male firefighters	Height: 177 ± 6 cm Weight: 77.2 ± 8.7 kgs BMI: 24.7 ± 2.1 kg·m^−2^ HR_max_: 90 ± 5% (176 ± 9 bmp) $\dot{v}$O2peak: 43.1 ± 4.9 mL·kg·min. Treadmill test	Incremental treadmill test, child rescue, 250m run, find an exit, 250 m run 2	Discrete testing 23 kgs	There was a significant correlation between $\dot{v}$O2peak and time to job completion of the simulated intervention (r = 0.09, *p* = 0.72). Correlation coefficients ranging from 0.09 to 0.53 existed between $\dot{v}$O2peak and time to complete the different tasks.
Phillips et al. [79]	longitudinal study, cohort and cross-sectional study design Canada, Alberta	414 male firefighter applicants	Age: 27 ± 5 years Height: 180 ± 6 cm Mass: 89.0 ± 17.0 kgs BMI: 26.9 ± 4.2 kg·m^−2^ Treadmill: 15.9 ± 2.7 min Treadmill test	Hose drag, Weighted sled pull Forcible entry, Victim rescue, Ladder climb	PPE only Sequential testing 23.3 kgs	There was a significant correlation between body mass and treadmill test duration and a stronger correlation (r = 0.76) between test duration and $\dot{v}$O2peak relative to total mass. The less than 70.0 and 70.0 to 79.9 kg mass categories were significantly slower compared with the others during the charged hose drag. For the weighted sled pull, forcible entry and victim rescue tasks, the less than 70 kg group was significantly slower. The more than 110.0 kg group was significantly slower than all the other groups on the ladder climb test. There were modest correlations (*p* < 0.05) between body mass and task completion time for the charged hose drag and weighted sled pull tests (r = 0.44, r = 0.36, respectively. There were weak correlations between task completion time and body mass for the forcible entry, victim rescue, and ladder climb tests.
Rhea et al. [42]	Cross-sectional study USA, Phoenix	20 male firefighters	Age: 34.5 ± 6.1 years Professional service: 6.1 ± 5.2 years BF%: 16.6 ± 3.9% 12-min run, bench press 5 rm(kg) squat 5 rm (kg), hand grip strength (kg), row endurance, bench press endurance, shoulder press endurance (reps), bicep curl endurance, squat endurance, ab curls, hand grip endurance, 400-m run, body fat %:	Hose pull Dummy drag Stair climb Hoist	Full SCBA gear Discrete testing 25 kg	Significant correlations (*p* < 0.05) between job performance and the following variables: total fitness (r = −0.62), bench press strength (r = −0.66), hand grip strength (r = −0.71), bent-over row endurance (r = −0.61), bench press endurance (r = −0.73), shoulder press endurance (r = −0.71), bicep endurance (r = −0.69), squat endurance (r = −0.47), and 400-m sprint time (r = 0.79). Significant correlations were also identified for each of the individual job performance tests.
Ryan et al. [69]	Cross-sectional study USA, North Carolina	41 full-time male firefighters	Age: 32.3 ± 2.5 years Stature: 178.3 ± 2.4 cm Body mass: 92.3 ± 5.7 kgs BMI: 29.0 ± 1.6 kg·m^−2^ BF%: 24.1 ± 2.4%	Stair climb time	Fitted with weighted vest to simulate weight of PPE. 22.73 kgs	Faster firefighter Stair Climb times (lower scores) were significantly associated with greater Peak Torque (r = −0.421; *p* = 0.007), greater PP (r = −0.530; *p* = 0.001), less fatigability (r = −0.389; *p* = 0.014), younger age (r = 0.441; *p* = 0.004), lower %BF (r = 0.629; *p* < 0.001).
Saari et al. [74]	Cross-sectional study USA, Ohio	74 full-time male firefighters	Younger vs. Older Age: 31.80 ± 3.42 vs. 44.65 ± 5.18 years Height: 179.85 ± 6.32 vs. 182.23 ± 5.57 cm Body mass: 92.61 ± 8.73 vs. 89.77 ± 23.06 kgs BF%: 15.94 ± 4.31 vs. 19.49 ± 4.58% Fat mass: 14.95 ± 4.84 vs. 17.71 ± 7.52 kgs Fat-free mass: 77.65 ± 6.32 vs. 72.06 ± 17.12 kgs Waist circumference: 88.67 ± 6.56 vs. 72.06 ± 17.12 cm Hip circumference: 102.47 ± 4.70 vs. 105.14 ± 6.57 cm	High-rise pack carry (stair climb) Hose hoist Forcible entry Hose advance Victim recue	Full PPE and SCBA gear. Sequential testing Not specified	On average, it took older firefighters 8.8% longer to complete the course compared with younger firefighters (*p* = 0.029,). Age was positively correlated with course time (*r* = 0.297, *p* = 0.017)
Schonfeld et al. [60]	Cross-sectional study USA	20 male volunteer firefighters	Age: 38.6 ± 2.5 years Height: 175.7 ± 1.1 cm Weight: 75.4 ± 1.9 kgs $\dot{v}$O2max: 48.5 ± 2.1 mL·kg·min BF%: 22.4 ± 0.9% Treadmill test	Stair climb Chopping simulation Victim drag	Full PPE and SCBA gear Sequential testing 24 kg	$\dot{v}$O2max (r = −0.628) and BF% (r = 0.467) were correlated with total performance time. BF% was only correlated individually to stair climb (r = 0.535), whereas $\dot{v}$O2max was correlated to all stair climb, chopping and victim drag (r = −0.627, −0.324 and −0.447)
Sheaff et al. [72]	Cross-sectional study USA, Baltimore–Washington	33 Career and volunteer firefighters	Age: 28 ± 1 years Height: 179.2 ± 1.6 cm Weight: 87.6 ± 3.8 kgs BMI: 27.1 ± 0.9 kg·m^−2^ BF%: 22.2 ± 1.1% $\dot{v}$O2_max_: 41.5 ± 1.4 mL·kg·min Cycle ergometer, treadmill test, chest press, leg press, knee extension.	Stair climb Hose drag Equipment carry Ladder raise and extension Forcible entry Search Rescue Ceiling breach and pull	Full SCBA gear Sequential testing CPAT 22.7 kgs	$\dot{v}$O2max (r = 20.602; *p* = 0.001), 4-finger isometric grip strength (r = 20.504; *p* = 0.009), and upper body strength (r = 20.485; *p* = 0.001) were also significantly related to CPAT performance. Furthermore, maximal HR response to stair climbing was significantly related to performance time (r = 0.523; *p* = 0.01), and percent of maximal HR during the stair climb (r = 0.488; *p* = 0.012).
Siddall et al. [28]	Cross-sectional study United Kingdom, London	68 (63 male; 5 female) full-time firefighters	Age: 41 ± 8 years Mass: 85.7 ± 12.9 kgs Height: 1.78 ± 0.06 m BF%: 19.7 ± 5.6% Fat mass: 17.3 ± 7.0 kgs absolute $\dot{v}$O2max: 4.0 ± 0.7 mL·kg·min relative $\dot{v}$O2max: 47.7 ± 9.0 mL·kg·min Treadmill test	The equipment carry: The casualty evacuation The ‘hose run’	Full PPE and SCBA gear Sequential testing 20.3 kgs	Relative $\dot{v}$O2 had a stronger inverse correlation with FFST performance time (R = −0.711; R2 = 0.506, SEE = ±56 s) than absolute $\dot{v}$O2 (R = −0.577; R2 = 0.332; SEE = ±65 s), explaining ~18% more of the variance in FFST performance. The combination of variables that produced the strongest prediction of FFST time was the absolute $\dot{v}$O2 and fat mass, which explained 26% and 8% of the variance.
Sothmann et al. [39]	Cross-sectional study USA, Chicago	153 full-time male and female firefighters	Age: 36 ± 6 years Years as firefighter: 8 ± 5 years Height: 172 ± 7.6 cm Weight: 84 ± 13 kgs	Hose drag and high rise pack carry Dummy drag	Discrete testing Not disclosed	Women completed the simulation approximately 35% slower than men which when tested by ANOVA proved to be a statistically significant difference (F 1151 = 5.70, *p* = 0.01). There was a significant age effect (F 3149 = 5.76, *p* < 0.01) on the performance times of the simulation protocol. Firefighters aged 50 years and over performed the protocol significantly slower than each of the three younger age classifications.
Stevenson et al. [64]	Cross-sectional study United Kingdom, London	69 full-time male and female firefighters	Age: 40 ± 8 years Mass: 85.8 ± 12.8 kgs Height: 178 ± 6 cm BMI: 27.0 ± 3.6 kg·m^−2^ BF%: 19.7 ± 5.5% $\dot{v}$O2max: 47.8 ± 9.0 mL·kg·min Treadmill test	Equipment carry Casualty evacuation Hose run	Full PPE and SCBA gear Sequential testing 20.2 kgs	The time to complete the firefighting simulation test (FFST) was highly inversely correlated with cardiorespiratory fitness (r = −0.73, *p* = 0.01).
Skinner et al. [30]	Cross-sectional study Australia	42 full-time male Aviation Rescue Firefighters	Age: 38.4 ± 7.6 years Height: 180.2 ± 6.6 cm Body mass: 81.9 kgs BMI: 26.2 ± 2.2 kg·m^−2^ Fat mass: 18.3 ± 5.6 kgs Lean mass: 62.7 ± 6.5 kgs BF%: 21.5 ± 4.6% $\dot{v}$O2max: 49.5 ± 6.9 mL·kg·min Treadmill test, 3rm bench press, 3rm leg press (kg), total grip strength (kg), anaerobic step test (max), sit and reach, abdominal curl, push ups.	*Simulated aircraft rescue and firefighting (ARFF) tasks* Hose drag (s) Dummy drag (s) Stihl saw hold (min) Stair climb (s)	Full SCBA gear Sequential testing 16.5 kgs	Older age, and longer arm length had small-to-moderate correlations with slower time to complete the dummy drag and hose drag tasks respectively. A strong inverse correlation was observed between time to complete the simulated ARFF emergency protocol for speed at lactate threshold, anaerobic step test performance and $\dot{v}$O2max. 3RM bench press presented a moderate to strong inverse correlation to hose drag performance time. The muscular endurance measure of maximal push-ups was significantly inversely correlated (r = −0.3) with hose drag performance time. A strong inverse correlation was observed between time to complete the simulated ARFF emergency protocol for speed at lactate threshold, anaerobic step test performance and $\dot{v}$O2max
von Heimburg et al. [71]	Cross-sectional study Norway, Trondheim	14 Part-time male firefighters	Age: 38 ± 9 years Height: 1.79 ± 0.07 m Weight: 83 ± 11 kgs BMI: 26 ± 2 kg·m^−2^ Waist circumference: 94 ± 7 cm Hip circumference: 102 ± 5 cm Waist-to-hip ratio: 0.92 ± 0.04 $\dot{v}$O2max: 4.4 ± 0.3 L·min $\dot{v}$O2max: 53 ± 5 mL·kg·min Treadmill test, leg press, bench press, press behind the neck.	Stair climb Six patient Victim drag	Full PPE and SCBA gear Sequential testing	The peak oxygen uptake in absolute terms was 18% higher in the faster subjects than in the slower ones during the rescue. The accumulated oxygen uptake obtained by integrating the oxygen uptake over the whole operation was less in the faster subjects, both in absolute terms (17%) and relative to body mass (25%). The faster firefighters had an 8% higher $\dot{v}$O2max expressed in absolute terms, but there was no difference between the two groups when the $\dot{v}$O2max was expressed relative to body mass. The eight faster subjects were stronger (13%) than the six slower ones in terms of the pooled strength index.
von Heimburg et al. [58]	Cross-sectional study Norway, Trondheim	22 full-time firefighters	23 Males/1 female Age: 42 ± 9 vs. 26 years Height: 1.82 ± 0.05 vs. 1.69 cm Body mass: 85 ± 9 vs. 58 kgs BF%: 23 ± 6% vs. 16% Lean body mass: 66 ± 6 kgs vs. 49 kgs BMI: 26 ± 2 kg·m^−2^ vs. 20.3 kg·m^−2^ NLIA treadmill test	Part 1: Puzzle Balance Hose drag Hose connection and disconnect Carrying heavy cans Tunnel crawling Part 2: Heat chamber Part 3: Retreat	Full PPE and SCBA gear Sequential testing 28 kgs	Firefighters with high $\dot{v}$O2max completed the test faster than firefighters with lower $\dot{v}$O2max. Performance on the Trondheim test correlated with the measured strength on all three strength tests and with the pooled strength index; the stronger participants were the fastest
von Heimburg et al. [70]	Cross-sectional study Norway, Trondheim	43 experienced and inexperienced male and female firefighters	Age: 41.4 ± 4.2 years Body mass: 84 ± 9.9 kgs Height: 1.81 ± 0.06 cm BMI: 25.5 ± 2.6 kg·m^−2^ BF%: 21.6 ± 5.8% LBM: 65.8 ± 5.9 kgs NLIA Tests	Trondheim test Part 1: Puzzle Balance Hose dragging Hose connection and disconnect Carrying heavy cans Tunnel crawling Part 2: Heat chamber Part 3: Retreat	Full PPE and SCBA gear Sequential testing 23 kgs	The young men performed the skill and agility tasks faster than the senior firefighters and the female applicants.
Williford et al. [77]	Cross-sectional study USA, Alabama	91 full-time male firefighters	Age: 31.69 ± 7.39 years Height: 177.29 ± 6.38 cm Weight: 83.97 ± 10.86 kgs BF%: 13.78 ± 4.31% $\dot{v}$O2 peak relative: 45.0 ± 6.0 mL·kg·min $\dot{v}$O2 peak absolute: 3.75 ± 0.43 L·min 1.5 mile run (s), Pull-ups, Push-ups Sit and reach(cm), Sit ups, Total grip strength (kg)	Victim rescue: 48.10 ± 29.36 Forcible entry: 30.44 ± 18.62 Hoist: 32.11 ± 21.87 Hose advance: 19.38 ± 18.88 Stair climb: 53.53 ± 13.68	Full PPE and SCBA gear Sequential testing 23 kgs	Significant correlations (*p* < 0.01) were found between the total obstacle course time and the following: total grip strength (r = −0.54), FFW (r = −0.47), height (r = −0.40), pull-ups (r = −0.38), push-ups (r = −0.38), 1.5 mile run (r = −0.38), sit-ups (r = −0.32), weight (r = −0.30) and BF% (r = 0.30. FFW and 1.5 mile run times to predict total obstacle course time (r = 0.71, r2 = 0.50, SE = 99.18 s).
Windisch, et al. [75]	Cross-sectional study Germany, Munich	41 full-time male firefighters	Age: 39 ± 9 years Height: 179.6 ± 2.3 cm Weight: 84.4 ± 9.2 kgs BMI: 26.1 ± 2.8 kg·m^−2^ $\dot{v}$O2max: 45.0 ± 6.0 mL·kg·min Treadmill test, leg press, hand grip, partial-curl ups, push-ups, shoulder press, rowing, standing long jump, sit and reach.	Ladder climb: 85 ± 15 s Hoist: 35 ± 8 s Crawling: 412 ± 96 s	Full PPE with SCBA gear and without SCBA gear Sequential tasks	It can be noted that outstanding performers had significantly higher $\dot{v}$O2 peak (*p* = 0.001) and significantly lower mean heart rates during REPE (*p* = 0.001) while completing the exercise faster (*p* = 0.001) compared to average, below average and poor performers. Aerobic fitness was a significant predictor of the speed a firefighter can perform the tasks
Xu et al. [63]	Cross-sectional study China, South East	20 full-time firefighters	Age: 25.65 ± 2.97 years; Height: 172.4 ± 4.8 cm; Body mass: 69.0 ± 8.9 kgs $\dot{v}$O2: 46.85 mL·kg·min BF%: 14.65% upper body muscular power: 675.35 watts lower body muscular power: 1705 watts Cycle ergometer, chest press, sitting leg power.	Rope climb Run 200 m round trip with load Run 60 m carrying a ladder Climb stairs with load Evacuation of 400 m with supplies Run 5 km with an air respirator and Run 100 m with a water hose		An increase in $\dot{v}$O2max decreased the time to complete firefighting tasks. Increased BF% increased the time to complete each task. Increased upper body strength the time to complete each task decreased. Increased lower body strength decreased the time to complete each task.

**Note:** Units of measurements: m—meters; cm—centimeters; kgs—kilograms; FFW—fat free weight; $\dot{v}$O2—oxygen consumption; $\dot{v}$O2max—maximum oxygen consumption; BF%—bodyfat percentage; kg·m^−2^—kilograms per meter squared; mL·kg·min.—milliliters per kilogram per minute; L·min—liters per minute; min—minutes; s—seconds; bmp—beats per minute. PPE—personal protective equipment; SCBA—self-contained breathing apparatus.

### 3.6. The Effect of Aging, Obesity, Heart Rate and Gender on Occupational Performance in Firefighters

**Figure 2:**
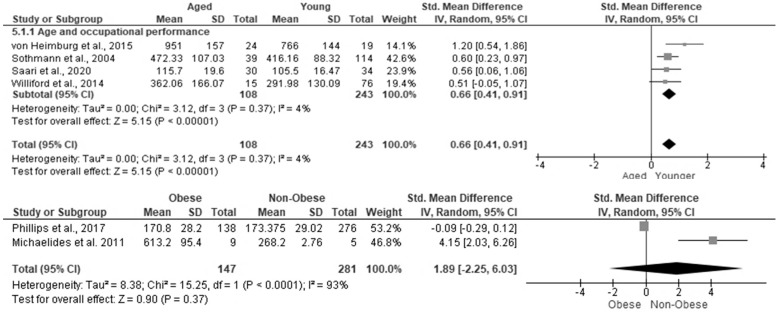
The effect of age and obesity on occupational performance in firefighters [39,57,70,74,77,79].

**Figure 3:**
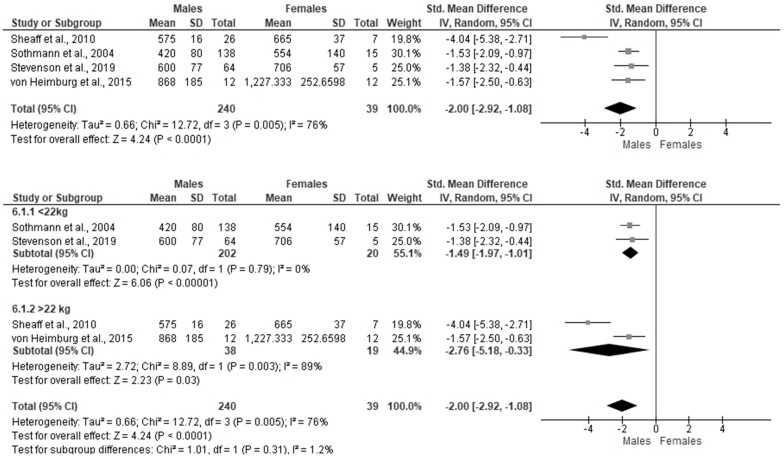
The effect of gender on occupational performance in firefighters. Subgroup analysis on the weight of PPE.

Figure 2 shows the effects of age and obesity on occupational performance in firefighters. Due to the different methods used to determine firefighters’ performance on occupational performance tasks, the standardized mean difference (SMD) was used to determine overall effect size. Age had a moderate significant pooled random effect on occupational performance [SMD = 0.66, 95%CI (0.41, 0.91), Z = 5.15, *p* < 0.001] [39,58,74,77]. The level of heterogeneity was low (I^2^ = 4%) and there was no evidence of publication bias (Egger test *p* = 0.397). For obesity, there was a large random effect size, that was not statistically significant [SMD = 1.89, 95%CI (−2.25, 6.03), Z = 0.90, *p* = 0.37; I^2^ = 93%] [57,79] (Figure 3).

Gender had a large effect size on occupational performance, indicating that males performed significantly better, which was statistically significant [SMD = −2.00, 95%CI (−2.50, −0.63), Z = 4.24, *p* < 0.001] [39,64,70,72,86] (Figure 3); with considerable heterogeneity between the studies, and no evidence of publication bias (*p* = 0.217 for the Egger test). In subgroup analysis according to the weight of PPE, there was no heterogeneity between studies that used equipment weighing less than 22 kgs in total (I^2^ = 0%); while the total effect decreased, the overall effect remained large [SMD = −1.49, 95%CI (−1.97, −1.01), Z = 6.06, *p* < 0.001].

### 3.7. Correlation between Obesity, Aging and Resting Heart Rate on Occupational Performance

In Table 4, there was a low positive correlation between BF% and occupational time (R = 0.316, *p* < 0.001) [30,42,57,60,65,73,74,77,80]. There was moderate heterogeneity between studies (I^2^ = 54.51%). In subgroups analyses, the correlation between BF% and completion time increased for all subgroups and was highest for the subgroups of males only (R = 0.413, *p* < 0.001) and full-time firefighters only (R = 0.388, *p* < 0.001). In addition, these subgroups had the least heterogeneity present (I^2^ ≤ 16.4%). There was a modest positive correlation between age and occupational performance (R = 0.224, *p* < 0.001) [30,57,65,66,69,74,77,80]. There was moderate-to-substantial heterogeneity present between studies (I^2^ = 74.1%). The correlation coefficient increased in studies that included either only male firefighters (R = 0.282, *p* < 0.001) or full-time firefighters (R = 0.323, *p* < 0.001) for the association between age and occupational performance. In addition, heterogeneity significantly decreased to 32.5% in male only studies and 0% in full-time firefighters’ studies. There was a low positive correlation between heart rate and occupational performance in firefighters (R = 0.387, *p* < 0.001), with no evidence of heterogeneity [57,73].

### 3.8. Correlation between Fitness Parameters and Occupational Performance

In Table 5, there was a significant moderate negative correlation between cardiorespiratory fitness and completion times (R = −0.584, *p* < 0.001) [28,30,58,60,64,65,66,68,70,72,80]; with substantial heterogeneity between the five studies (I^2^ = 72.9%). In subgroup analysis, studies where cardiorespiratory fitness was determined through gas analysis, and studies that only included male firefighters were more homogenous (I^2^ = 0.0% and I^2^ < 9.9%). However, the strongest correlation was present between studies that included only gas analysis to determine cardiorespiratory fitness (R = −0.672, *p* < 0.001). Upper body endurance had a significant low negative correlation with completion times (R = −0.344, *p* < 0.001; I^2^ = 0%) [30,57,66,70,73,77,80]. After subgroup analysis the highest correlation (R = −0.363, *p* < 0.001) [30,57,66,70,73,77] was present between upper body endurance and completion times studies where the weight of PPE was over 22 kgs. There was a significant low negative correlation between abdominal endurance and completion times (R = −0.308, *p* < 0.001; I^2^ = 0%) [30,42,57,65,66,73,77,80]. 

For strength, there was a significant low negative correlation between grip strength and completion times (R = −0.421, *p* < 0.001; I^2^ = 68.6%) [30,42,57,72,77,80]. Subgroup analysis did not explain the heterogeneity between studies; however, the highest correlation between grip strength and completion times when the weight of PPE was above 22 kgs (R = −0.473, *p* < 0.001). There was a significant low correlation between upper body strength and completion times (R = −0.318, *p* < 0.001; I^2^ = 57.7%) [30,42,57,66,72,73,80]. In subgroup analysis studies that included five or more tasks that were sequential were more homogenous (I^2^ = 5.1%). The highest correlation was found between upper body strength and occupational performance where studies only included male firefighters (R = −0.374, *p* < 0.001). Lower body strength had a significant negligible negative correlation with completion times (R = −0.216, *p* = 0.020; I^2^ = 0%) [42,57,73].

### 3.9. Correlation between Obesity and Age on Individual Task Performance in Firefighters

In Table 6, there was a significant low positive correlation between BF% and stair climb times (R = 0.489, *p* < 0.001; I^2^ = 39.2%) [30,42,57,60,76,77]. In subgroup analysis there was no heterogeneity between studies where the weight of PPE was above 22 kgs and where five or more tasks were performed. In addition, there was a moderate positive correlation between BF% and stair climb times (R = 0.514, *p* < 0.001) [42,57,60,76,77] when the weight of PPE was more than 22 kgs, and when five or more tasks were performed (R = 0.537, *p* < 0.001) [57,77]. There was a significant low positive correlation between BF% and hose drag time (R = 0.241, *p* < 0.001) [30,42,57,60,77], between BF% and victim rescue (R = 0.254, *p* < 0.001) [30,42,57,60,77], BF% and forcible entry (R = 0.285, *p* < 0.001) [77,86], and between BF% and equipment hoist (R = 0.197, *p* = 0.041) times [42,77]. There was no heterogeneity for the studies included in the meta-analysis for the hose drag, victim rescue and equipment hoist. The highest correlation was present between BF% and hose drag when the weight of PPE was 22 kgs or above (R = 0.255, *p* < 0.001) [42,57,60,77]. For forcible entry, moderate heterogeneity was present between studies. There was a significant low correlation between age and stair climb time (R = 0.345, *p* < 0.001; I^2^ = 62.3%) [30,69,76,77]. After subgroup analysis on studies including only full-time firefighters there was 0.0% heterogeneity present.

For age, there was a low positive correlation between age and stair climb times (R = 0.345, *p* < 0.001; I^2^ = 62.3%) [30,69,76,77]. After subgroup analysis, 0.0% heterogeneity was present when studies that analysed full-time male firefighters only were included. In addition, the correlation was strongest between age and stair climb times when studies that included only full-time male firefighters were analysed (R = 0.434, *p* < 0.001) [30,69,77].

### 3.10. Correlation between Physical Fitness and Individual Task Performance

In Table 7, there was a significant low negative correlation between cardiorespiratory fitness and stair climb times (R = −0.421, *p* = 0.004; I^2^ = 82.9%) [30,31,61,65]. After subgroup analysis, there was a significant moderate negative correlation between cardiorespiratory fitness and stair climb times (R = −0.513, *p* < 0.001) [31,61,65], but considerable heterogeneity remained. There was a significant negative correlation between cardiorespiratory fitness and victim rescue (R = −0.320, *p* = 0.003; I^2^ = 57.1%) [30,61,65,66] and between cardiorespiratory fitness and hose drag times (R = −0.197, *p* = 0.046; I^2^ = 38.1%) [30,31,66]. In subgroup analysis, there was no heterogeneity between studies where the weight of PPE was above 22 kgs for victim rescue and hose drag. 

There was a significant low negative correlation between upper body endurance and stair climb times (R = −0.408, *p* < 0.001; I^2^ = 0.0%) [30,57,77] (Table 7). Subgroup analysis was performed on equipment weighing over 22 kgs, which increased the strength of the correlation between studies (R = −0.436, *p* < 0.001) [57,77]. There were significant low negative correlations between upper body endurance and hose drag times (R = −0.260, *p* < 0.001; I^2^ = 0.0%) [30,57,66,77], victim rescue times (R = −0.200, *p* = 0.026; I^2^ = 55.2%) [30,57,66,77] and forcible entry times (R = −0.247, *p* = 0.006; I^2^ = 51.1%) [57,66,77]. There was homogeneity between studies investigating upper body endurance and hose drag times, and moderate heterogeneity present between upper body endurance and victim rescue and forcible entry times (I^2^ = 55.2% and I^2^ = 51.1%, respectively). Subgroup analysis did not explain the heterogeneity between studies. However, there was no evidence of publication bias present for victim rescue (Egger’s test *p* = 0.536) or forcible entry (Egger’s test *p* = 0.109).

There was a significant low negative correlation between abdominal endurance and stair climb times (R = −0.415, *p* < 0.001; I^2^ = 25.7%) [30,42,57,61,77] (Table 6). After subgroup analysis there was no heterogeneity present where the weight of PPE over 22 kgs (I^2^ = 0.0%) and five or more tasks were performed (I^2^ = 0.0%). In addition, the correlation was highest for the studies where the weight of PPE was above 22 kgs (R = −0.452, *p* < 0.001) [42,57,61,77] and five or more tasks were performed (R = −0.472, *p* < 0.001) [57,61,77]. There were significant negligible negative correlations between abdominal endurance and hose drag times (R = −0.230, *p* < 0.001; I^2^ = 17.3%) [30,42,57,66,77], between abdominal endurance and victim rescue times (R = −0.119, *p* = 0.039; I^2^ = 41.4%) [30,42,57,61,66,77] and between abdominal endurance and forcible entry times (R = −0.195, *p* = 0.002; I^2^ = 0.0%) [57,66,77]. After subgroup analysis, heterogeneity was reduced (I^2^ = 0.0% and I^2^ = 29.1%, respectively), for hose drag and victim drag times when controlling for studies that used five or more tasks and tasks that were performed sequentially.

There was a significant low negative correlation between grip strength and hose drag times (R = −0.378, *p* = 0.005) [30,31,42,57,61,77]. There was substantial heterogeneity present between studies (I^2^ = 78.9%), without evidence of publication bias (Eggar’s test *p* = 0.379). After subgroup analysis where five or more tasks were performed, heterogeneity was reduced (I^2^ = 42.5%) [57,77], but moderate heterogeneity remained. The highest correlation (R = −0.442, *p* = 0.005) [31,42,57,77] was present between grip strength and hose drag time where the weight of PPE was more than 22 kgs. There was a significant moderate negative correlation between grip strength and victim rescue time (R = −0.578, *p* < 0.001) [30,31,42,57,77], with substantial heterogeneity between studies (I^2^ = 68.2%). After subgroup analysis, heterogeneity was reduced (I^2^ = 35.5%) when full-time male firefighters only were included [30,42,57,77]. In addition, after subgroup analysis, there was a moderate negative corelation between grip strength and victim rescue (R = −0.609, *p* = 0.049) [42,57,61,77] when equipment weighed more than 22 kgs. There were significant low negative correlations between grip strength and forcible entry times (R = −0.426, *p* = 0.001; I^2^ = 67.2%) [57,77] and between grip strength and equipment hoist times (R = −0.420, *p* = 0.039; I^2^ = 64.8%) [57,77]. 

There was a significant moderate negative correlation between upper body strength and hose drag times (R = −0.544, *p* = 0.001) [30,31,42,57], with substantial heterogeneity present (I^2^ = 71.9%). After subgroup analysis there was no heterogeneity present (I^2^ = 0.0%) [30,57], where five or more sequential tasks were performed. In addition, after subgroup analysis, performed there was a moderate negative correlation between upper body strength and victim rescue times (R = −0.609, *p* = 0.049) [42,57] when the weight of PPE was more than 22 kgs. However, considerable heterogeneity was present (I^2^ = 85.9%). There was a significant low negative correlation between upper body strength and victim rescue times (R = −0.350, *p* = 0.012; I^2^ = 56.1%) [30,42,57]. After subgroup analysis, no heterogeneity was present (I^2^ = 0.0%) [30,57], when five or more sequential tasks were performed. 

There were significant low negative correlations between lower body strength and hose drag times (R = −0.244, *p* = 0.001) [30,31,42,57], and between lower body strength and victim rescue times (R = −0.254, *p* = 0.004) [30,42,57], with studies being homogenous. There was a significant negligible negative correlation between flexibility and stair climb times (R = −0.190, *p* = 0.030) [30,77], with low heterogeneity present between studies (I^2^ = 11.4%).

## 4. Discussion

### 4.1. Summary of Evidence

The results of this systematic review and meta-analysis indicated that the effect of cardiovascular risk status and musculoskeletal health status on occupational performance are understudied, and large gaps exist in the literature. Only two cardiovascular disease risk factors were frequently studied, namely age and obesity, and both had a significant effect on occupational performance. The results indicated that as firefighters aged and accumulated more adipose tissue, their completion times increased, which was consistent for all tasks investigated. In addition, we found a significant effect of physical fitness on occupational performance with cardiorespiratory fitness, muscular endurance, and upper body strength, all related to all individual tasks performance. These results are consistent with two systematic reviews, one on firefighters and the other on military personnel, that also found that aerobic capacity, muscular endurance and muscular strength are related to completion times in emergency occupations [87,88]. In addition, the current study results indicated that the weight of PPE worn significantly influenced the performance of all tasks. Moreover, the weight of PPE was related to overall occupational performance and individual task performance according to age, obesity and all physical fitness measures. This may be due to the weight of the equipment placing an extra burden on firefighters’ abilities to perform their tasks efficiently, especially when compounded with excessive adipose accumulation and older age. The weight of PPE may become particularly important when conducting occupational performance tasks, as using full PPE may represent the truest simulation of the burden firefighters face physiologically while on active duty. These results are supported by a systematic review that indicated that the weight of PPE and SCBA gear elicit a significant physiological response in firefighters [32].

Globally, firefighting is regarded as one of the most physically demanding occupations that require high levels of physical fitness in order for them to perform their jobs effectively [88]. Moreover, firefighters are expected to remain in peak physical conditioning, especially as they age, to ensure they do not become a liability as they remain in the fire services [88,89]. The results of the current review supported this standpoint, as less physically fit firefighters that had increased fat mass were the most likely to perform poorly on the occupational performance tasks. Firefighting induces significant physiological responses [9,33] and, therefore, fitter firefighters perform significantly better than unfit firefighters, even as they age.

### 4.2. The Effect of Age, Obesity, Blood Pressure, Heart Rate and Gender on Occupational Performance

The results indicated that age had a significant moderate effect on occupational performance in the current study. In addition, a significant correlation existed between aging and overall occupational performance, particularly among full-time career firefighters. Ageing is considered a CAD risk factor, particularly in men 45 years and older and woman 55 years and older, due to the progressive reduction in arterial elasticity, increased inflammatory responses and reduction in key growth factors responsible for maintenance of arterial health [90,91,92,93]. Moreover, diastolic blood pressure was shown to significantly affect occupational performance in firefighter, however, the literature on this is limited, and more research should be conducted to allow for meta-analysis. Previous research indicated that blood pressure significantly affected work capacity in athletes [94] and job performance in emergency personnel [95] alike, which supports the results of the current study. Regular physical activity maintains cardiovascular health, however, firefighters generally become less physically active as they age [96,97,98,99], particularly in firefighters in the City of Cape Town Fire and Rescue Service [15,22]. Firefighters that are older, especially those aged 45 years or older, should engage in regular physical activity to maintain their work performance to acceptable standards [96,97,99,100]. There was a significant positive correlation between age and stair climb performance. Older firefighters performed significantly worse compared to younger firefighters and showed the strongest correlation when occupational performance simulation protocols included five or more sequential tasks. Age did not correlate with hose drag, victim rescue and forcible entry performance. The results suggest that muscular endurance and strength are of greater significance in performing the hose drag, victim rescue and forcible entry tasks successfully. Aging had a much larger effect on cardiorespiratory fitness as opposed to muscular endurance and strength, which may explain why aged firefighters performed worse on the stair climb [37,38,96,99,100,101]. The present results indicated that cardiorespiratory fitness was the most significant factor in optimal performance in firefighters, and that older firefighters with lower cardiorespiratory fitness had the lowest overall occupational performance, particularly those that are obese [38,99,102,103,104]. A study by Von Heimburg [71] reported that firefighters that performed best on the hose drag had a better dragging technique and higher cardiorespiratory fitness, but no significant difference between age was present. The years of experience as a firefighter may, somewhat, reduce the effect of age on task performance, especially those tasks where economical and explosive technique, rather than absolute power, may prove to be most beneficial, such as hose drag, victim rescue and forcible entry. 

Obesity had a significant large moderate effect on occupational performance in firefighters, indicating that non-obese firefighters performed significantly better on the occupational performance tasks. This was further strengthened by the correlation analysis which indicated that as firefighters’ age increased, overall simulation performance significantly decreased, and in particular, the stair climb, and victim drag events, especially when the weight of PPE was controlled for. Obesity increases the amount of non-functional excess weight that firefighters are required to overcome while performing their duties, reducing their overall performance on simulated tasks [99,102,103,104,105]. Although research has indicated that increased body mass, to a point, may benefit certain strength or upper body stamina related tasks, overall task performance was not benefited, particularly related to the stair climb task [71,79]. Obese firefighters, generally, have a much lower cardiorespiratory fitness level, which may account for the reduced occupational performance seen in this group [26,27,103,106]. To maintain high work performance, firefighters should maintain a healthy weight throughout their careers, especially those firefighters involved in smoke diving and emergency rescues [3,6,8,107]. Although there were no studies investigating other CVD risk factors, obesity has been associated with increased risk status. Reducing obesity may not only improve overall occupational performance, but may also reduce all-cause mortality related to CVD in firefighters [2,3,6,16,108]. Increased adiposity reduced the overall performance times in stair climb, hose drag, victim rescue, forcible entry, and equipment hoist times in firefighters. Firefighters that were obese, performed significantly worse on each task. Most firefighting tasks were negatively affected by increased fat mass in firefighters, which is consistent with previous research indicating that obesity reduces performance [87]. 

Resting heart rate had a significant positive correlation with completion times, indicating that a higher resting heart rate resulted in worse performance on the occupational performance tasks. Resting heart rate (RHR) is closely linked to cardiorespiratory health and cardiorespiratory fitness. Higher RHRs have been linked to cardiovascular disease and poor cardiorespiratory fitness and increased cardiovascular risk [27,106,109]. Nazari et al. [33] reported that high heart rates and near maximum heart rates are reached during occupational performance tasks. 

The current results indicated that gender had a significant effect on completion times in firefighters, with males performing significantly better than female firefighters. This is consistent with previous results that indicated males were stronger and fitter than their female counterparts and performed the occupational tasks faster. This may be due to many tasks being strength and endurance based, favouring male firefighters [31,86]. This is most likely due to males being taller, more muscular, and stronger than female firefighters, which has been shown to be a significant predictor of performance times [79]. Female firefighters may need to engage in more frequent off-duty strength training to maintain the minimum levels of strength needed to perform firefighting tasks optimally.

### 4.3. The Effect of Physical Fitness on Occupational Performance

The results indicated that a moderate negative correlation existed between cardiorespiratory fitness and completion times. Fitter firefighters performed significantly better on the occupational performance tasks compared to less fit firefighters. Studies suggest that firefighting require a minimum $\dot{V}$O2max of 42 mL·kg·min and, unsurprisingly, firefighters with higher cardiorespiratory fitness levels performed significantly better. This is supported by Hauschild et al. [88], where the review indicated that emergency personnel that had higher cardiorespiratory fitness performed better in the simulated tasks. Although all physical fitness parameters, except flexibility, was significantly correlated to occupational performance, cardiorespiratory fitness had the highest correlation with overall performance. Maintenance of cardiorespiratory fitness may be the most important aspect in the maintenance of optimal work performance in firefighters. This is especially true when firefighters that find themselves in emergency situations and are required to work at moderate-to-vigorous levels of intensity for prolonged periods of time. Cardiorespiratory fitness was significantly and negatively correlated to stair climb and hose drag times, especially when subgroup analysis was performed on studies including heavier equipment weights (>22 kgs). The stair climb and hose drag tasks require firefighters to perform locomotive move either climbing a flight of stairs or dragging a hose, which require the use of large muscle groups that require large amounts of oxygen. Fitter firefighters are able to utilize the available oxygen more efficiently, performing better on these locomotive tasks. Heavier equipment increased the cardiorespiratory load of each firefighting task, and require a higher fitness level for adequate completion [32].

Upper body (push-up) and abdominal (sit-ups) endurance had a significant negative correlation with overall completion times, particularly when firefighters performed five or more tasks and when equipment weighed more than 22 kgs. Many of the tasks’ firefighters are required to perform involve forceful repetitive upper body exertive movements. Higher levels of upper body muscular endurance allow firefighters to sustain a particular amount of force over a number of repetitions [61,62,66,77]. Such as the door breach, which require firefighters to sustain maximal force during each hit to move the tyre or sled the desired distance [57,66,110]. Significant negative correlations were present between upper body and abdominal endurance and stair climb, hose drag, victim rescue, and forcible entry performance and, in particular, when subgroup analysis was performed on studies with equipment weighing more than 22 kgs and five or more tasks. Higher levels of upper body and abdominal stamina positively affected performance in stair climb, hose drag, victim rescue and forcible entry tasks. For all tasks, firefighters are required to wear their full protective equipment and SCBA gear which places significant strain on the upper body muscular [79,111,112]. Higher levels of upper body endurance will reduce the muscular strain of wearing PPE and SCBA gear while performing the occupational tasks. As indicated by Marcel-Millet [9], there are significant physiological differences between firefighters that wore PPE and SCBA gear, compared to those without. Focussing on improving firefighter stamina may prove to be particularly important to maintain high levels of occupational performance.

Grip strength, upper body strength and lower body strength were all significantly and negatively correlated with overall simulation performance in firefighters, particularly in males, where five or more tasks were performed while wearing equipment weighing more than 22 kgs. In general, stronger firefighters completed the simulation protocols significantly quicker than weaker firefighters. As mentioned previously, stronger firefighters are capable of producing higher levels of force with each movement, as most studies indicated significant relationships existed between muscle strength and endurance in firefighters [42,57,61,62]. In addition, higher levels of strength reduce the effort required to perform each task, allowing them to sustain the minimum required level of force for longer. This allows firefighters to move the tyre or sled further with each swing of the sledgehammer, or hoisting equipment further with each pull. More specifically, grip strength correlated negatively to hose drag, victim rescue, forcible entry and equipment hoist times, upper and lower body strength was negatively correlated to hose drag and victim rescue times, only, and in particular, when heavier equipment was used. Surprisingly, lower body strength was not correlated with better performance in the stair climb task. Grip strength appeared to be the most significant strength measure to maintain overall occupational performance in firefighters. This may be due to firefighting requiring firefighters to constantly grip and hold objects in place while producing high levels of force, such as sledgehammers, axes, jaws of life and fire hoses [42,61,62,77]. Higher levels of upper and lower body strength may allow firefighters to carry and drag the hose and victim with less effort [61,62,65,87]. There were insufficient studies available to analyse the effect of upper and lower body strength on forcible entry or equipment hoist times. 

Flexibility was the only physical fitness parameter that was not significantly correlated to overall occupational performance, however, was negatively correlated to stair climb times in firefighters. More flexible firefighters may be able to have longer strides while climbing the stairs, as the hamstring is able to stretch further with less discomfort, improving the stair climb performance. A systematic review reported that hamstring flexibility was a key factor sprinting, jumping and agility [113]. Although the present study did not find a significant correlation between flexibility and other firefighter tasks, maintenance of flexibility may assist in maintaining high levels of occupational performance in firefighters [57,66,73]. Importantly, higher flexibility has been shown to reduce the incidence of injury in firefighters [43,44,114].

### 4.4. Limitations of the Study

The large number of cross-sectional studies are a limitation of the current study. Heterogeneity was introduced due to differences in weight of equipment and age ranges of the firefighters across different studies. However, this was at least partially mitigated through subgroup analysis. A limited number of studies were conducted on the relationship between cardiovascular and musculoskeletal health and occupational performance, which negatively impacted the meta-analysis on these variables. The older studies included in this systematic review, may have influenced the results, as advancements in PPE, work environments and intervention techniques, have in different physical demands, compared with previous years. Limitations in the quality of evidence are described below.

### 4.5. Applicability of Evidence

The results indicated that non-obese, younger male firefighters that have a high cardiorespiratory fitness level, and those that have high levels of muscular endurance and strength have the most favorable overall occupational performance. Cardiorespiratory fitness, along with upper body and abdominal endurance should be prioritized in exercise training programmes. Moreover, tasks that were performed sequentially where the weight of PPE worn was over 22 kgs significantly and negatively affected overall performance times. Taller, heavier male firefighters may have the most favorable performance outcomes when performing occupational specific duties. An inherent limitation of the evidence is that all firefighters recruited to participate in the studies are apparently healthy and injury free. More studies need to be conducted on firefighters with cardiovascular disease risk factors and underlying musculoskeletal health issues. Regular aerobic training, along with strength training may prove to be particularly beneficial for older firefighters who are smaller in stature and have a lower body mass, and in particular, female firefighters.

### 4.6. Quality of Evidence

Critical appraisal of the included studies for the majority of studies were acceptable; however, few studies appraised were low in overall quality. Three studies scored 15 points in the AXIS appraisal too, but was largely due to the small sample sizes of the studies. Due to the difficulty of performing occupational simulation tasks, most studies included small number of firefighters to participate. High heterogeneity was present between approximately half of the analysis, and possibly due to the difference is sample sizes, which may have influenced the means, standard deviations, as well the correlation strength between the included variables. Although high heterogeneity was present, the studies provided valuable information on factors affecting occupational performance.

### 4.7. Gaps in the Literature

The effects of cardiovascular disease risk factors and musculoskeletal health on firefighters’ occupational performance are understudied. Particularly, studies related to the effect of cardiovascular risk factors, such as hypertension, diabetes, dyslipidaemia, and cigarette smoking on occupational performance, and the effect of musculoskeletal health issues on occupation performance. More research should be conducted on cardiovascular risks and musculoskeletal health related to occupational performance in firefighters.

### 4.8. Implications for Future Research

More research should be conducted investigating the effect of cardiovascular disease risk factors, and overall risk status on occupational performance. The effect of musculoskeletal health on work performance is also understudied.

## 5. Conclusions

Age and obesity significantly affected occupational performance in firefighters, increasing task completion times across all events. Physical fitness is integral to occupational performance in firefighters, with cardiorespiratory fitness, muscular endurance and upper body strength having the most significant effect on total completion times and all individual tasks. The weight of PPE is an essential consideration, as this significantly impacts completion times, highlighted by the heterogeneity caused between studies, particularly when five or more tasks were performed sequentially while firefighters wore PPE weighing over 22 kg. Moreover, younger, stronger, and heavier male firefighters performed significantly better than older, lighter and weaker firefighters, which emphasizes the importance of maintaining a suitable body composition, and appropriate levels of muscular endurance and strength as firefighters age. Firefighting departments should adopt regular physical activity, focused on maintaining cardiorespiratory fitness, muscular endurance, and upper body strength, to maintain firefighters’ physical fitness and dietary recommendations, to reduce the likelihood of overweight and obesity in firefighters, which is particularly important as they age.

## 6. Patents

### Protocol Registration

Details of the protocol for this systematic review were registered on PROSPERO (CRD42021258898) and can be accessed at: https://www.crd.york.ac.uk/prospero/display_record.php?RecordID=258898 (accessed on 21 January 2022).

## Figures and Tables

**Table 1:** Critical appraisal of cross-sectional studies using AXIS checklist.

Question	Michaelides et al. [57]	Skinner et al. [30]	von Heimburg et al. [58]	Perroni et al. [59]	Schonfeld et al. [60]	Lindberg et al. [61]	Lindberg et al. [62]	Siddal et al. [28]	Xu et al. [63]	Stevenson et al. [64]	Myhre et al. [65]	Chizewski et al. [66]	Kleinberg et al. [67]	Elsner and Kolkhorst [68]	Ryan et al. [69]	von Heimburg et al. [70]	von Heimburg et al., [71]	Rhea et al. [42]	Sheaff et al. [72]	Michaelides et al. [73]	Saari et al. [74]	Windisch, et al. [75]	Misner et al. [76]	Williford et al. [77]	Davis et al. [78]	Nazari et al. [31]	Sothmann et al. [39]
Introduction																											
Clear aims/objectives	🗸	🗸	🗸	🗸	🗸	🗸	🗸	🗸	🗸	🗸	🗸	🗸	🗸	🗸	🗸	🗸	🗸	🗸	🗸	🗸	🗸	🗸	🗸	🗸	🗸	🗸	🗸
Methods																											
Study design appropriate for the stated aim(s)?	🗸	🗸	🗸	🗸	🗸	🗸	🗸	🗸	🗸	🗸	🗸	🗸	🗸	🗸	🗸	🗸	🗸	🗸	🗸	🗸	🗸	🗸	🗸	🗸	🗸	🗸	🗸
Sample size justified?	⨯	⨯	⨯	⨯	⨯	⨯	⨯	⨯	⨯	⨯	⨯	🗸	🗸	🗸	🗸	🗸	⨯	🗸	🗸	🗸	🗸	⨯	🗸	🗸	🗸	🗸	🗸
Target/reference population clearly defined?	🗸	🗸	🗸	🗸	🗸	🗸	🗸	🗸	🗸	🗸	🗸	🗸	🗸	🗸	🗸	🗸	🗸	🗸	🗸	🗸	⨯	🗸	🗸	🗸	🗸	🗸	🗸
Sample frame taken from an appropriate population base to closely represent the target population?	⨯	⨯	⨯	⨯	⨯	⨯	⨯	⨯	⨯	⨯	🗸	⨯	⨯	⨯	⨯	⨯	⨯	⨯	⨯	⨯	⨯	⨯	⨯	🗸	🗸	🗸	🗸
Selection process likely to select subjects that were representative of the target population?	⨯	⨯	⨯	⨯	⨯	⨯	⨯	⨯	⨯	⨯	🗸	⨯	⨯	⨯	⨯	⨯	⨯	⨯	⨯	⨯	🗸	⨯	⨯	🗸	🗸	🗸	🗸
Measures undertaken to address and categorize non-responders?	⨯	⨯	⨯	⨯	⨯	⨯	⨯	🗸	🗸	🗸	⨯	🗸	🗸	🗸	🗸	🗸	🗸	🗸	🗸	🗸	🗸	🗸	🗸	🗸	🗸	🗸	🗸
Were the risk factor and outcome variables measured appropriate to the aims of the study?	🗸	🗸	🗸	🗸	🗸	🗸	🗸	🗸	🗸	🗸	🗸	🗸	🗸	🗸	🗸	🗸	🗸	🗸	🗸	🗸	🗸	🗸	🗸	🗸	🗸	🗸	🗸
Were the risk factor and outcome variables measured correctly using instruments/measurements that had been trialed, piloted or published previously?	🗸	🗸	🗸	🗸	🗸	🗸	🗸	🗸	🗸	🗸	🗸	🗸	🗸	🗸	🗸	🗸	🗸	🗸	🗸	🗸	🗸	🗸	🗸	🗸	🗸	🗸	🗸
Clear which tests were used to determine statistical significance and/or precision estimates?	🗸	🗸	🗸	🗸	🗸	🗸	🗸	🗸	🗸	🗸	🗸	🗸	🗸	🗸	🗸	🗸	🗸	🗸	🗸	🗸	🗸	🗸	🗸	🗸	🗸	🗸	🗸
Were the methods sufficiently described to enable them to be repeated?	🗸	🗸	🗸	🗸	🗸	🗸	🗸	🗸	🗸	🗸	🗸	🗸	🗸	🗸	🗸	🗸	🗸	🗸	🗸	🗸	🗸	🗸	🗸	🗸	🗸	🗸	🗸
Results																											
Were the basic data adequately described?	🗸	🗸	🗸	🗸	🗸	🗸	🗸	🗸	🗸	🗸	🗸	🗸	🗸	🗸	🗸	🗸	🗸	🗸	🗸	🗸	🗸	🗸	🗸	🗸	🗸	🗸	🗸
Does the response rate raise concerns about non-response bias?	🗸	🗸	🗸	🗸	🗸	🗸	🗸	🗸	🗸	🗸	🗸	🗸	🗸	🗸	🗸	🗸	🗸	🗸	🗸	🗸	🗸	🗸	🗸	🗸	🗸	🗸	🗸
If appropriate, was information about non-responders described?	N/A	N/A	N/A	N/A	N/A	N/A	N/A	N/A	N/A	N/A	N/A	N/A	N/A	N/A	N/A	N/A	N/A	N/A	N/A	N/A	N/A	N/A	N/A	N/A	N/A	N/A	N/A
Were the results internally consistent?	🗸	🗸	🗸	🗸	🗸	🗸	🗸	🗸	🗸	🗸	🗸	🗸	🗸	🗸	🗸	🗸	🗸	🗸	🗸	🗸	🗸	🗸	🗸	🗸	🗸	🗸	🗸
Were the results presented for all the analyses described in the methods?	🗸	🗸	🗸	🗸	🗸	🗸	🗸	🗸	🗸	🗸	🗸	🗸	🗸	🗸	🗸	🗸	🗸	🗸	🗸	🗸	🗸	🗸	🗸	🗸	🗸	🗸	🗸
Discussion																											
Were the authors’ discussions and conclusions justified by the results?	🗸	🗸	🗸	🗸	🗸	🗸	🗸	🗸	🗸	🗸	🗸	🗸	🗸	🗸	🗸	🗸	🗸	🗸	🗸	🗸	🗸	🗸	🗸	🗸	🗸	🗸	🗸
Were the limitations of the study discussed?	🗸	🗸	🗸	🗸	🗸	🗸	🗸	🗸	🗸	🗸	⨯	🗸	🗸	🗸	🗸	🗸	🗸	🗸	🗸	🗸	🗸	🗸	🗸	🗸	🗸	🗸	🗸
Other																											
Were there any funding sources or conflicts of interest that may affect the authors’ interpretation of the results?	🗸	🗸	🗸	🗸	🗸	🗸	🗸	🗸	🗸	🗸	🗸	🗸	🗸	🗸	🗸	🗸	🗸	🗸	🗸	🗸	🗸	🗸	🗸	🗸	🗸	🗸	🗸
Was ethical approval or consent of participants attained?	🗸	🗸	🗸	🗸	🗸	🗸	🗸	🗸	🗸	🗸	🗸	🗸	🗸	🗸	🗸	🗸	🗸	🗸	🗸	🗸	🗸	🗸	🗸	🗸	🗸	🗸	🗸
TOTAL SCORE	15	15	15	15	15	15	15	16	16	16	16	17	17	17	17	17	17	17	17	17	17	17	18	19	19	19	19

Note: NA—indicated questions that were not applicable for grading of the overall study quality; 🗸—indicates yes; ⨯—indicates no.

**Table 2:** Critical appraisal of cohort studies using the Critical Appraisal Skills Programme checklist.

Quality Assessment Criteria	Phillips et al. [79]	MacDermid et al. [29]	Hendersen et al. [80]
1. Did the study address a clearly focused issue?	🗸	🗸	🗸
2. Was the cohort recruited in an acceptable way?	🗸	🗸	🗸
3. Was the exposure accurately measured to minimize bias?	🗸	🗸	🗸
4. Was the outcome accurately measured to minimize bias?	🗸	🗸	🗸
5. (a) Have the authors identified all important confounding factors?	🗸	🗸	🗸
5. (b) Have they taken account of the confounding factors in the design and/or analysis?	🗸	🗸	🗸
6. (a) Was the follow up of subjects complete enough?	🗸	🗸	🗸
6. (b) Was the follow up of subjects long enough?	🗸	🗸	🗸
7. What are the results of this study?	NA	NA	NA
8. How precise are the results?	NA	NA	NA
9. Do you believe the results?	🗸	🗸	🗸
10. Can the results be applied to the local population?	🗸	🗸	🗸
11. Do the results of this study fit with other available evidence?	🗸	🗸	🗸
12. What are the implications of this study for practice?	🗸	🗸	🗸
TOTAL SCORE	12	12	12

Note: NA—indicated questions that were not applicable for grading of the overall study quality; 🗸—indicates yes.

**Table 4:** The correlation between age, obesity and heart rate and occupational task performance in firefighters.

Outcome	No. of Studies	No. of Participants	R (95% CI)	Z Score	*p* (Overall Effect)	Heterogeneity I^2^; Cohen’s Q; *p*	Egger’s Test Intercept (95%CI); *p*	Begg’s Test (τ_;_ *p*)
Age	8	944	0.224 (0.162 to 0.284)	3.834	<0.001 **	74.1%; 27.0136; <0.001	2.33 (−2.36–7.02); 0.269	0.18; 0.529
*Five or more tasks*	5	639	0.199 (0.0425 to 0.346)	2.484	0.004 **	71.1%; 13.85; 0.008	4.18 (−2.14–10.48); 0.126	0.53; 0.197
*Males only*	6	416	0.282 (0.167 to 0.390)	4.675	<0.001 **	32.5%; 7.41; 0.191	2.82 (−5.30–10.95); 0.389	0.41; 0.243
*Weight*	5	749	0.286 (0.0968 to 0.455)	2.927	0.003 **	83.9%; 24.90; <0.001	3.69 (−6.39–13.77); 0.329	0.20; 0.624
*Full-time only*	5	327	0.323 (0.220 to 0.418)	5.912	<0.001 **	0.0%; 3.68; 0.452	1.47 (−0.70–9.95); 0.621	0.40; 0.327
Obesity	9	876	0.316 (0.254 to 0.375)	6.432	<0.001 **	54.5%; 17.59; 0.025	1.79 (−0.49–4.07); 0.106	0.14; 0.597
*Five or more tasks*	5	572	0.350 (0.184 to 0.496)	4.007	<0.001 **	71.8%; 14.18; 0.007	3.48 (−1.66–8.62); 0.120	0.60; 0.142
*Males only*	6	348	0.413 (0.319 to 0.498)	7.933	<0.001 **	0.0%; 5.65; 0.463	0.19 (−3.53–3.91); 0.901	0.09; 0.758
*Sequential testing*	6	614	0.368 (0.218 to 0.501)	4.601	<0.001 **	69.1%; 16.19; 0.006	3.43 (−0.01–6.88); 0.051	0.47; 0.189
*Weight of PPE*	7	770	0.354 (0.228 to 0.468)	5.253	<0.001 **	62.4%; 15.94; 0.014	1.72 (−1.44–4.89); 0.220	0.19; 0.538
*Full-time only*	6	512	0.388 (0.310 to 0.460)	9.095	<0.001 **	16.4%; 5.98; 0.308	0.54 (−3.21–4.28); 0.712	0.33; 0.348
Heart rate	2	110	0.398 (0.226 to 0.547)	4.301	<0.001 **	0.0%; 0.41; 0.521	2.74 (--); <0.001	1.00; 0.317

**Note:** ** indicates statistical significance <0.01. (--)—indicates insufficient studies to calculate Egger’s test result. PPE—personal protective equipment.; italics—indicates subgroup analysis.

**Table 5:** The correlation between physical fitness and occupational performance in firefighters.

Outcome	No. of Studies	No. of Participants	R (95% CI)	Z Score	*p* (Overall Effect)	Heterogeneity I^2^; Cohen’s Q; *p*	Egger’s Test Intercept (95%CI); *p*	Begg’s Test (τ_;_ *p*)
Cardiorespiratory fitness	11	946	−0.584 (−0.671 to −0.482)	−9.132	<0.001 **	72.9%; 36.96; <0.001	−2.52 (−4.80 to 0.23); 0.034	−0.18; 0.432
Gas analysis	5	207	−0.672 (−0.743 to −0.587)	−11.295	<0.001	0.0%; 3.98; 0.407	1.17 (−3.72 to 6.07); 0.501	0.40; 0.327
Sequential tasks	8	635	−0.589 (−0.682 to −0.476)	−8.390	<0.001 **	64.1%; 19.49; 0.007	−2.05 (−4.65 to 0.53); 0.099	−0.29; 0.322
Five or more tasks	6	525	−0.571 (−0.680 to −0.438)	−7.074	<0.001 **	61.9%; 13.11; 0.022	−1.99 (−4.88 to 0.91); 0.129	−0.33; 0.348
Males	7	281	−0.596 (−0.675 to −0.505)	−10.260	<0.001 **	9.9%; 6.66; 0.353	−0.72 (−4.04 to 2.59); 0.599	−0.19; 0.538
Males and females	4	665	−0.566 (−0.709 to −0.378)	−5.161	<0.001 **	88.2%; 25.46; <0.001	−7.31 (−15.92 to 1.29); 0.067	−0.33; 0.497
Weight of PPE	7	678	−0.551 (−0.660 to −0.419)	−7.005	<0.001 **	68.3%; 18.94; 0.004	−2.15 (−4.61 to 0.31); 0.075	−0.09; 0.758
Full-time only	7	449	−0.605 (−0.729 to −0.443)	−6.094	<0.001 **	75.0%; 24.0390; <0.001	−2.39 (−0.60 to 1.25); 0.152	0.00; 1.000
Upper body endurance	6	387	−0.344 (−0.430 to −0.251)	−6.886	<0.001 **	0.0%; 3.49; 0.624	1.13 (−4.07 to 6.33); 0.579	0.33; 0.348
Weight of PPE	4	256	−0.363 (−0.467 to −0.250)	−5.949	<0.002 **	0.0%; 1.3; 0.770	−7.6 (−8.63 to 7.11); 0.717	−0.33; 0.497
Full−time only	4	268	−0.324 (−0.430 to −0.209)	−5.318	<0.001 **	1.9%; 3.06; 0.383	4.30 (−9.22 to 17.82); 0.305	0.33; 0.497
Abdominal endurance	8	871	−0.308 (−0.367 to −0.246)	−9.256	<0.001 **	0.0%; 3.62; 0.822	−0.05 (−1.65 to 1.54); 0.939	0.14; 0.621
Five or more tasks	5	587	−0.333 (−0.403 to −0.258)	−8.267	<0.001 **	0.0%; 2.22; 0.696	−0.9 (−3.33 to 3.13); 0.929	0.00; 1.000
Sequential tasks	5	254	−0.320 (−0.428 to −0.202)	−5.121	<0.001 **	0.0%; 1.60; 0.808	1.38 (−1.35 to 4.11); 0.206	0.60; 0.142
Males only	5	323	−0.349 (−0.443 to −0.247)	−6.391	<0.001 **	0.0%; 2.01; 0.733	2.24 (−1.36 to 5.8); 0.142	0.60; 0.142
Weight of PPE	5	740	−0.296 (−0.361 to −0.229)	−8.212	<0.001 **	0.0%; 2.38; 0.795	0.09 (−1.89 to 2.09); 0.089	0.07; 0.851
Full-time only	5	446	−0.294 (−0.377 to −0.205)	−6.284	<0.001 **	0.0%; 1.82; 0.768	−0.52 (−3.22 to 2.18); 0.584	0.00; 1.000
Grip strength	6	258	−0.421 (−0.602 to −0.198)	−5.086	<0.001 **	68.6%; 15.92; 0.007	0.59 (−4.30 to 5.48); 0.754	−0.07; 0.851
Five or more tasks	4	502	−0.439 (−0.578 to −0.274)	−4.882	<0.001 **	68.9%; 9.67; 0.022	1.39 (−9.64 to 12.42); 0.642	0.67; 0.174
Males only	5	258	−0.421 (−0.602 to −0.198)	−3.542	<0.001 **	71.1%; 13.84; 0.008	0.59 (−430 to 5.48); 0.754	−0.07; 0.851
Weight of PPE	5	522	−0.473 (−0.604 to −0.317)	−5.420	<0.001 **	66.9%; 12.07; 0.017	−0.11 (−6.30 to 6.09); 0.959	−0.20; 0.624
Full-time only	4	225	−0.406 (−0.625 to −0.127)	−2.790	0.005 **	77.5; 13.31; 0.004	−1.38 (−21.05 to 18.29); 0.791	0.00; 1.000
Upper body strength	8	814	−0.318 (−0.380 to −0.254)	−5.756	<0.001 **	57.7%; 16.53; 0.207	−1.51 (−4.29 to 1.27); 0.232	−0.29; 0.320
Five or more tasks	5	530	−0.374 (−0.446 to −0.298)	−8.931	<0.001 **	5.1; 4.21; 0.378	−1.26 (−4.36 to 1.85); 0.288	−0.20; 0.624
Sequential tasks	6	572	−0.357 (−0.428 to −0.283)	−8.802	<0.001 **	28.7; 7.01; 0.219	−0.42 (−3.82 to 2.98); 0.750	−0.06; 0.851
Males only	6	286	−0.421 (−0.540 to −0.266)	−5.183	<0.001 **	42.4%; 8.68; 0.122	−0.83 (−6.93 to 5.28); 0.726	−0.20; 0.573
Weight of PPE	6	683	−0.339 (−0.449 to −0.219)	−5.321	<0.001 **	50.2; 10.04; 0.074	−1.89 (−4.52 to 0.74); 0.116	−0.60; 0.091
Full-time only	5	389	−0.313 (−0.470 to −0.137)	−3.409	0.001 **	57.5%; 9.40; 0.052	−2.40 (−6.17 to 1.37); 0.136	−0.80; 0.050
Lower body strength	3	122	−0.216 (−0.383 to −0.0349)	−2.331	0.020 *	0.0%; 0.27; 0.876	−0.22 (−12.99 to 12.55); 0.863	−0.33; 0.602
Five or more tasks	2	102	−0.201 (−0.383 to −0.003)	−1.992	0.046 *	0.0%; 0.10; 0.749	1.01 (--); <0.001	1.00; 0.317
Full-time only	2	92	−0.236 (−0.424 to −0.029)	−2.232	0.026 *	0.0%; 0.10; 0.751	−0.70 (--); <0.001	−1.00; 0.317
Flexibility	4	233	−0.099 (−0.227 to 0.032)	−1.479	0.139	0.0%; 2.05; 0.560	−2.58 (−8.81 to 3.64); 0.216	−0.67; 0.174

**Note:** * indicates statistical significance <0.05; ** indicates statistical significance <0.01. (--)—indicated insufficient studies present to perform Egger’s test. PPE—personal protective equipment; italics – indicates subgroup analysis.

**Table 6:** The correlation between obesity and age, and individual task performance.

Outcome	No. of Studies	No. of Participants	R (95% CI)	Z Score	*p* (Overall Effect)	Heterogeneity I^2^; Cohen’s Q; *p*	Egger’s test Intercept (95%CI); *p*	Begg’s Test (τ_;_ *p*)
Obesity								
Stair climb	6	304	0.489 (0.361 to 0.599)	6.696	<0.001 **	39.2%; 8.23; 0.144	−1.98 (−6.92 to 2.97); 0.33	−0.28; 0.44
*Five or more tasks*	2	160	0.537 (0.416 to 0.640)	7.453	<0.001 **	0.0%; 0.12; 0.729	3.43 (--); <0.001	1.00; 0.317
*Sequential tasks*	4	222	0.485 (0.375 to 0.581)	7.670	<0.001 **	36.8%; 4.75; 0.191	−1.26 (−12.73 to 10.21); 0.682	−0.33; 0.497
*Males only*	5	242	0.468 (0.308 to 0.577)	7.654	<0.001 **	33.6%; 6.02; 0.197	−1.78 (−7.33 to 3.78); 0.383	−0.32; 0.439
*Weight of PPE*	4	200	0.514 (0.401 to 0.611)	7.789	<0.001 **	0.0%; 2.07; 0.557	−1.29 (−6.31 to 3.73); 0.385	−0.18; 0.709
*Full-time only*	4	222	0.435 (0.259 to 0.583)	4.543	<0.001 **	48.9%; 5.8771; 0.118	−3.45 (−11.86 to 4.96); 0.219	−0.33; 0.497
Hose drag	5	242	0.241 (0.095 to 0.378)	3.580	<0.001 **	19.5%; 4.97; 0.290	1.54 (−3.59 to 6.66); 0.411	0.53; 0.197
*Five or more tasks*	2	160	0.231 (−0.004 to 0.442)	1.926	0.054	55.8%; 2.26; 0.133	14.85 (--); <0.001	1.00; 0.317
*Sequential tasks*	4	222	0.249 (0.0702 to 0.412)	2.710	0.007 **	39.6%; 4.97; 0.174	2.46 (−7.39 to 12.31); 0.395	0.67; 0.174
*Weight of PPE*	4	200	0.255 (0.117 to 0.383)	3.577	<0.001 **	31.5%; 4.38; 0.223	1.81 (−5.65 to 9.26); 0.407	0.55; 0.264
*Full-time only*	4	222	0.206 (0.073 to 0.3310	3.022	0.003 **	0.0%; 2.59; 0.458	0.27 (−8.63 to 9.18); 0.908	0.33; 0.497
Victim drag	5	242	0.254 (0.129 to 0.371)	3.915	<0.001 **	0.0%; 1.51; 0.825	−0.35 (−3.51 to 2.81); 0.746	−0.11; 0.796
*Five or more tasks*	2	160	0.280 (0.129 to 0.419)	3.575	<0.001 **	0.0%; 0.22; 0.639	4.52 (--); <0.001	1.00; 0.317
*Sequential tasks*	4	222	0.244 (0.113 to 0.366)	3.601	<0.001 **	0.0%; 1.15; 0.765	−1.45 (−5.45 to 2.56); 0.261	−0.33; 0.497
*Males only*	5	242	0.254 (0.129 to 0.371)	3.915	<0.001 **	0.0%; 1.51; 0.825	−0.35 (−3.51 to 2.81); 0.746	−0.11; 0.796
*Weight of PPE*	4	200	0.275 (0.138 to 0.401)	3.864	<0.001 **	0.0%; 0.99; 0.805	−0.16 (−4.53 to 4.2); 0.886	0.18; 0.709
*Full-time only*	4	222	0.266 (0.136 to 0.386)	3.946	<0.001 **	0.0%; 1.08; 0.782	0.38 (−5.27 to 6.03); 0.801	0.00; 1.000
Forcible entry	2	160	0.285 (0.135 to 0.423)	3.639	<0.001 **	24.1%; 0.1.32; 0.251	11.51 (--); <0.001	1.00; 0.317
Equipment hoist	2	111	0.197 (0.008 to 0.372)	2.044	0.041 *	0.0%; 0.65; 0.419	−1.58 (--); <0.001	−1.00; 0.317
Age								
Stair climb	4	324	0.345 (0.166 to 0.502)	3.669	<0.001 **	62.3%; 7.74; 0.052	2.72 (−9.94 to 15.38); 0.453	0.33; 0.497
*Sequential tasks*	2	133	0.431 (0.280 to 0.562	5.201	<0.001 **	7.7; 1.08; 0.298	−3.74 (--); <0.001	−1.00; 0.317
*Full-time male firefighters*	3	174	0.434 (0.302 to 0.549)	5.963	<0.001 **	0.0%; 1.09; 0.581	−2.26 (−27.04 to 22.53); 0.454	−0.33; 0.602
Hose drag	3	222	0.0403 (0.094 to 0.173)	0.589	0.556	0.0%; 0.26; 0.889	0.46 (−19.37 to 20.29); 0.817	−0.33; 0.602
Victim rescue	3	222	0.147 (−0.079 to 0.359)	1.280	0.200	62.8%; 5.37; 0.068	6.62 (−44.75 to 57.99); 0.349	0.33; 0.602
Forcible entry	2	180	0.0318 (−0.116 to 0.178)	0.419	0.675	0.0%; 0.08; 0.771	35.74 (--); <0.001	1.00; 0.317

**Note:** * indicates statistical significance <0.05; ** indicates statistical significance <0.01. (--)—indicates insufficient studies to calculate Egger’s test result. PPE—personal protective equipment.; italics—indicates subgroup analysis.

**Table 7:** The correlation between physical fitness and individual task performance.

Outcome	No. of Studies	No. of Participants	R (95% CI)	Z Score	*p* (Overall Effect)	Heterogeneity I^2^; Cohen’s Q; *p*-Value	Egger’s test Intercept (95%CI); *p*	Begg’s Test (τ_;_ *p*)
**Cardiorespiratory fitness**								
Stair climb	4	351	−0.421 (−0.639 to −0.140	−2.856	0.004 **	82.9%; 17.55;<0.001	4.39 (−4.24 to 13.03); 0.159	0.33; 0.497
*Sequential testing*	3	302	−0.451 (−0.702 to −0.100)	−2.472	0.013 *	85.4%; 13.69; 0.001	−8.39 (−254.21 to 237.44); 0.739	−0.33; 0.602
*Weight of PPE*	3	309	−0.513 (−0.680 to −0.296)	−4.244	<0.001 **	70.7%; 6.81; 0.033	3.13 (−23.58 to 29.83); 0.377	0.33; 0.602
*Full-time only*	2	91	−0.214 (−0.406 to −0.005)	−2.007	0.045 *	5.8%; 1.06; 0.303	17.68 (--); <0.001	1.00; 0.317
Hose drag	3	180	−0.197 (−0.376 to −0.004)	−1.997	0.046 *	38.1%; 3.23; 0.198	3.64 (−55.99 to 63.26); 0.580	1.00; 0.117
*Five or more and Sequential*	2	131	−0.138 (−0.415 to 0.163)	−0.897	0.370	61.9%; 2.62; 0.105	5.98 (--); <0.001	1.00; 0.317
*Weight of PPE*	2	138	−0.278 (−0.427 to −0.114)	−3.279	0.001 **	0.0%; 0.04; 0.839	0.93 (--); <0.001	−1.00; 0.317
Victim drag	4	391	−0.356 (−0.500 to −0.194)	−4.146	<0.001 **	57.1; 6.99; 0.072	2.09 (−6.87 to 11.05); 0.421	0.33; 0.497
*Five or more tasks*	2	127	−0.384 (−0.525 to −0.223)	−4.450	<0.001	0.0%; 0.69; 0.406	−2.72 (--); <0.001	−1.00; 0.317
*Sequential tasks*	3	169	−0.300 (−0.504 to −0.066)	−2.488	0.013 *	55.6%; 4.50; 0.105	0.88 (−73.51 to 75.27); 0.905	−0.33; 0.602
*Males only*	2	131	−0.220 (−0.482 to 0.079)	−1.449	0.147	61.6%; 2.60; 0.107	5.95 (--); <0.001	1.00; 0.317
*Weight of PPE*	2	260	−0.452 (−0.544 to −0.349)	−7.757	<0.001 **	0.0%; 0.05; 0.817	−4.41 (--); <0.001	−1.00; 0.317
*Full-time only*	3	353	−0.320 (−0.501 to −0.113)	−2.977	0.003 **	69.3%; 6.61; 0.039	4.37 (−6.36 to 15.14); 0.122	1.00; 0.117
Saw hold	2	80	0.301 (−0.601 to 0.074)	−1.580	0.114	64.8%; 2.84; 0.092	−44.09 (--); <0.001	−1.00; 0.317
**Upper body endurance**								
Stair climb	3	205	−0.408 (−0.518 to −0.285)	−6.061	<0.001 **	0.0%; 1.28; 0.527	3.93 (−9.22 to 17.07); 0.164	1.00; 0.1172
*Weight of PPE*	2	163	−0.436 (−0.553 to −0.301)	−5.850	<0.001 **	0.0%; 0.37; 0.541	7.13 (--); <0.001	1.00; 0.3173
Hose drag	4	294	−0.290 (−0.393 to −0.180)	−5.010	<0.001 **	0.0%; 0.56; 0.905	−2.15 (−6.03 to 1.72); 0.139	−0.33; 0.497
*Weight of PPE*	2	205	−0.290 (−0.413 to −0.157)	−4.183	<0.001 **	0.0%; 0.56; 0.754	0.78 (--); <0.001	1.00; 0.317
*Full-time only*	3	163	−0.266 (−0.404 to −0.115)	−3.410	0.001 **	0.0%; 0.00; 0.947	−2.48 (−15.86 to 10.89); 0.256	−0.33; 0.602
Victim rescue	4	294	−0.200 (−0.363 to −0.025)	−2.23	0.026 *	55.2%; 6.69; 0.083	4.01 (−19.23 to 27.24); 0.536	0.67; 0.174
*Weight of PPE*	2	163	−0.197 (−0.537 to 0.197)	−0.980	0.327	84.5%; 6.44; 0.011	29.60 (--); <0.001	1.00; 0.317
*Full-time only*	3	205	−0.183 (−0.420 to 0.077)	−1.383	0.167	69.9; 6.63; 0.036	4.30 (−99.57 to 108.17); 0.692	0.33; 0.602
Forcible entry	3	252	−0.247 (−0.407 to −0.072)	−2.743	0.006 **	51.1%; 4.09; 0.129	21.43 (−25.53 to 68.39); 0.109	1.00; 0.117
*Weight of PPE and full-time*	2	163	−0.220 (−0.488 to 0.086)	−1.411	0.158	74.3%; 3.88; 0.049	22.98 (--); <0.001	1.00; 0.317
**Abdominal endurance**								
Stair climb	5	262	−0.415 (−0.512 to −0.306)	−6.933	<0.001 **	25.7%; 5.38; 0.250	1.51 (−5.21 to 8.22); 0.526	0.00; 1.00
*Five or more tasks and sequential*	3	200	−0.472 (−0.574 to −0.354)	−7.079	<0.001 **	0.0%; 1.12; 0.572	−3.07 (−21.12 to 14.99); 0.276	−1.00; 0.117
*Full-time Males firefighters*	3	224	−0.388 (−0.496 to −0.268)	−5.962	<0.001 **	22.8%; 3.88; 0.274	2.53 (−5.17 to 10.23); 0.293	0.33; 0.497
*Weight of PPE*	4	220	−0.452 (−0.554 to −0.338)	−7.035	<0.001 **	0.0%; 2.52; 0.473	4.73 (−49.98 to 59.44); 0.470	0.33; 0.602
Hose drag	5	313	−0.230 (−0.334 to −0.120)	−4.034	<0.001 **	17.3%; 4.83; 0.305	2.61 (−2.40 to 7.62); 0.196	0.40; 0.327
*Five or more tasks*	3	251	−0.253 (−0.367 to −0.132)	−4.029	<0.001 **	0.0%; 1.05; 1.000	−5.72 (−116.29 to 104.85); 0.629	−0.33; 0.602
*Sequential tasks*	4	293	−0.256 (−0.361 to −0.143)	−4.381	<0.001 **	0.0%; 1.06; 0.786	−0.92 (−10.99 to 9.14); 0.732	0.00; 1.00
*Weight of PPE*	2	182	−0.157 (−0.367 to 0.068)	−1.374	0.169	48.8%; 3.91; 0.142	3.11 (25.36 to 31.59); 0.397	0.33; 0.602
*Full-time firefighters*	4	224	−0.201 (−0.326 to −0.069)	−2.961	0.003 **	27.9%; 4.16; 0.245	2.41 (−6.16 to 10.97); 0.350	0.33; 0.497
Victim drag	6	351	−0.151 (−0.290 to −0.006)	−2.044	0.041 *	41.4%; 8.52; 0.129	1.01 (−5.24 to 7.27); 0.677	0.33; 0348
*Five or more Tasks*	4	289	−0.189 (−0.342 to −0.027)	−2.276	0.023	46.6%; 5.62; 0.132	−3.96 (−21.81 to 13.89); 0.441	0.00; 1.00
*Sequential tasks*	5	331	−0.176 (−0.281 to −0.068)	−3.165	0.002 **	29.1%; 5.64; 0.228	−2.37 (−11.97 to 7.22); 0.489	0.00; 1.00
*Males only*	4	271	−0.113 (−0.231 to 0.008)	−1.834	0.067	34.3%; 4.57; 0.206	3.33 (-.345 to 10.12); 0.169	1.00; 0.042
*Weight of PPE*	4	220	−0.137 (−0.366 to 0.108)	−1.098	0.272	64.7%; 8.50; 0.037	1.16 (−14.11 to 16.44)	0.33; 0.497
*Full-time*	4	224	−0.0845 (−0.248 to 0.084)	−0.984	0.325	30.4%; 4.31; 0.230	2.58 (−5.77 to 10.93); 0.315	0.67; 0.174
Forcible entry	3	251	−0.195 (−0.313 to −0.072)	−3.081	0.002 **	0.0%; 1.39; 0.499	11.35 (−35.85 to 58.56); 0.201	0.33; 0.602
*Weight of PPE*	2	162	−0.160 (−0.308 to −0.004)	−2.012	0.044 *	0.0%; 0.79; 0.374	9.78 (--); <0.001	1.00; 0.317
Equipment hoist	2	111	−0.168 (−0.400 to 0.167)	−0.844	0.399	37.1%; 1.59; 0.207	2.48 (--); <0.001	1.00; 0.317
Saw hold	2	80	0.252 (−0.300 to −0.677)	−0.891	0.373	83.8%; 6.17; 0.013	64.96 (--); <0.001	1.00; 0.317
**Grip strength**								
Stair climb	6	312	−0.200 (−0.502 to 0.071)	−1.510	0.131	85.5%; 34.49;<0.0001	−2.29 (−15.69 to 11.10); 0.348	−0.33; 0.348
Hose drag	5	274	−0.378 (−0.589 to −0.119)	−2.806	0.005 **	78.9%; 19.00;<0.001	−3.97 (−16.26 to 8.33); 0.379	0.00; 1.00
*Five or more tasks*	2	163	−0.325 (−0.496 to −0.129)	−3.188	0.001 **	42.5%; 1.74; 0.188	15.38 (--); <0.001	1.00; 0.317
*Males only*	4	225	−0.429 (−0.668 to −0.108)	−2.566	0.010 *	83.3%; 17.92; <0.001	−3.69 (−24.39 to 17.01); 0.524	0.00; 1.00
*Weight of PPE*	4	232	−0.442 (−0.666 to −0.145)	−2.834	0.005 **	81.8%; 16.45;<0.001	−5.37 (−21.79 to 11.06); 0.295	−0.33; 0.497
Victim rescue	5	263	−0.578 (−0.713 to −0.402)	−5.545	<0.001 **	68.2%; 12.56; 0.014	−2.11 (−12.47 to 8.25); 0.563	−0.40; 0.327
*Five or more tasks*	3	201	−0.610 (−0.773 to −0.372)	−4.366	<0.001 **	78.8%; 9.43; 0.009	−6.89 (−95.23 to 81.44); 0.503	−0.33; 0.602
*Sequential tasks*	4	243	−0.561 (−0.716 to −0.353)	−4.697	<0.001 **	74.7%; 11.84; 0.008	−2.44 (−29.09 to 24.22); 0.732	−0.33; 0.497
*Full-time male firefighters*	4	225	−0.507 (−0.600 to −0.401)	−8.152	<0.001 **	35.5; 4.65; 0.199	−0.51 (−12.29 to 11.27); 0.869	0.00; 1.00
*Weight of PPE*	4	221	−0.621 (−0.758 to −0.432)	−5.388	<0.001 **	69.6%; 9.86; 0.019	−3.07 (−17.08 to 10.93); 0.445	−0.33; 0.497
Forcible entry	2	163	−0.426 (−0.623 to −0.179)	−3.248	0.001 **	67.2; 3.05; 0.081	20.36 (--); <0.001	1.00; 0.317
Equipment hoist	2	111	−0.420 (−0.703 to −0.023)	−2.066	0.039 *	64.8%; 2.84; 0.092	3.29 (--); <0.001	1.00; 0.317
Saw hold	2	80	0.468 (−0.0836 to 0.800)	1.682	0.093	85.1%; 6.70; 0.009	67.71 (--); <0.001	1.00; 0.317
**Upper body strength**								
Stair climb	3	134	−0.140 (−0.306 to 0.035)	−1.571	0.116	0.0%; 1.45; 0.484	−2.33 (−17.66 to 12.99); 0.304	−1.00; 0.117
Hose drag	3	134	−0.544 (−0.748 to −0.247)	−3.337	0.001 **	71.9%; 7.11; 0.029	−5.71 (19.89 to 8.48); 0.123	−1.00; 0.117
*Five or more tasks*	2	114	−0.402 (−0.547 to −0.233)	−4.421	<0.001 **	0.0%; 0.45; 0.502	−3.38 (--); <0.001	−1.00; 0.317
*Weight of PPE*	2	92	−0.609 (−0.888 to −0.002)	−1.966	0.049 *	85.9%; 7.10; 0.008	−5.91 (--); <0.001	−1.00; 0.317
Victim rescue	3	134	−0.350 (−0.573 to −0.080)	−2.512	0.012 *	56.1%; 4.55; 0.103	−3.10 (−47,24 to 41.04); 0.536	−0.33; 0.602
*Five or more tasks*	2	114	−0.255 (−0.422 to −0.073)	−2.715	0.007 **	0.0%; 0.67; 0.412	4.13 (--); <0.001	1.00; 0.317
*Weight of PPE*	2	92	−0.461 (−0.733 to −0.064)	−2.248	0.025 *	64.6%; 2.82; 0.093	−3.72 (--); <0.001	−1.00; 0.317
**Lower body strength**								
Stair climb	5	329	−0.0460 (−0.155 to 0.064)	−0.817	0.414	0.0%; 1.54; 0.819	−0.64 (−3.45 to 2.17); 0.522	−0.20; 0.624
Hose drag	4	179	−0.244 (−0.381 to −0.097)	−3.223	0.001 **	0.0%; 1.72; 0.632	−1.92 (−8.87 to 5.03); 0.357	0.00; 1.00
*five or more tasks*	2	110	−0.222 (−0.395 to −0.033)	−2.298	0022 *	0.0%; 0.29; 0.591	2.71 (--); <0.001	1.00; 0.317
*Weight of PPE*	3	137	−0.271 (−0.422 to −0.104)	−3.139	0.002 **	0.0%; 1.29; 0.525	−2.19 (−20.28 to 15.90); 0.367	−0.33; 0.602
Victim rescue	3	130	−0.254 (−0.411 to −0.081)	−2.851	0.004 **	0.0%; 0.29; 0.862	−0.93 (−11.45 to 9.60); 0.462	−0.33; 0.602
*five or more tasks*	2	110	−0.246 (−0.416 to −0.059)	−2.559	0.010 *	0.0%; 0.25; 0.619	−2.79 (--); <0.001	−1.00; 0.317
*Weight of PPE*	2	88	−0.229 (−0.422 to −0.017)	−2.111	0.035 *	0.0%; 0.13; 0.724	−0.81 (--); <0.001	−1.00; 0.317
**Flexibility**								
Stair climb	2	133	−0.190 (−0.351 to −0.019)	−1.959	0.030 *	11.4%; 1.13; 0.288	3.82 (--); <0.001	1.00; 0.317
Hose drag	3	222	−0.130 (−0.259 to 0.004)	−1.908	0.056	0.0%; 0.94; 0.626	−2.55 (−28.08 to 22.98); 0.425	−1.00; 0.012
Victim rescue	3	222	−0.0792 (−0.210 to 0.055)	−1.159	0.247	0.0%; 1.67; 0.434	−4.09 (−22.07 to 13.89); 0.212	−1.00; 0.117
Forcible entry	2	180	−0.0700 (−0.215 to 0.078)	−0.924	0.355	0.0%; 0.66; 0.418	99.59 (--); <0.001	1.00; 0.317

**Note:** * indicates statistical significance <0.05; ** indicates statistical significance <0.01. (--)—indicates insufficient studies to calculate Egger’s test result. PPE—personal protective equipment.; italics—indicates subgroup analysis.

## Data Availability

All data generated or analyzed during this study are included in the published review article.

## Supplementary Materials

The following supporting information can be downloaded at: https://www.mdpi.com/article/10.3390/ijerph191911946/s1, Supplementary S1: Search strategy for databases; Supplementary S2: Eligibility screening form; Supplementary S3: Data extraction form.
