# Supplementary material for: Effects of Cardiovascular Disease Risk Factors, Musculoskeletal Health, and Physical Fitness on Occupational Performance in Firefighters—A Systematic Review and Meta-Analysis
Source: Int J Environ Res Public Health. 2022 Sep 21;19(19):11946. doi: 10.3390/ijerph191911946 (PMC9564707; doi:10.3390/ijerph191911946)
Supplement: Supplementary file 1 [file ijerph-19-11946-s001.zip › Supplementary S1.pdf]

Appendix 1

Search syntax in different databases

PubMed:

#1 “firefighter” OR “fire and rescue personnel” OR “fire fighters” OR “fire fighter” OR “firefight”

#2 "cardiovascular system"[MeSH] OR ("cardiovascular" [All Fields] AND "system" [All Fields]) OR "cardiovascular system" [All Fields] OR "cardiovascular*" [All Fields] OR "cardiovascular abnormalities" [MeSH] OR “HRV” [All Fields] OR “heart rate variability” [All Fields] OR “Heart Rate Interval” [All Fields] OR “RR variability” [All Fields] OR “cycle length variability” [All Fields] OR “heart period variability” [All Fields] OR “autonomic function” [All Fields] OR “vagal control” [All Fields] OR “lipid profile” [All Fields] OR “cholesterol” [MeSH] OR “dyslipidaemia” OR “hypercholesteremia” OR “diabetes” AND “mellitus” OR “blood glucose” OR “age” OR “obesity” OR “hypertension” OR “blood pressure” OR “metabolic syndrome” OR “hyperglycaemia”

#3 “muscular injury” OR (“musculoskeletal” [All Fields] AND “system” [All Fields]) OR “muscular pain” OR “chronic pain” [All Fields] OR “acute pain” [All Fields] “acute injury” [All Fields] OR “muscular health” OR “muscular disorder”

#4 "physical fitness"[MeSH] OR “exercise” [All Fields] OR “physical exertion” [All Fields] OR “muscular strength” OR “muscular endurance” OR “aerobic fitness” OR “cardiorespiratory fitness” OR “cardiorespiratory capacity” OR “$\dot{V}O_2max$” OR “aerobic fitness” OR “power”

#5 “work performance” [All Fields] OR “endurance” [All Fields] OR “fitness” [All Fields] OR "work performance" [MeSH] AND "work classification" [All Fields] OR "occupational health" [MeSH] OR “employee health” OR “health, industrial” OR “industrial health” OR “occupational safety” OR “safety, occupational” OR “simulated work tasks” OR “simulated firefighting” OR “CPAT” OR “physical ability test”

#6 (#1 AND #2) OR (#1 AND #3) OR (#1 AND #4) OR (#1 AND #5) OR (#1 AND #2 AND #3) OR (#1 AND #2 AND #4) OR (#1 AND #3 AND #4) OR (#1 AND #2 AND #3 AND #4) OR (#1 AND #2 AND #3 AND #4 AND #5)

Scopus

#1 (TITLE-ABS-KEY “firefighter*”) + OR AND TITLE-ABS-KEY ("fire and rescue" OR firefighters OR fire fire fighter OR firefight OR firemen)

#2 AND ("cardiovascular" OR “cardiovascular abnormalities” OR “HRV*” OR “heart rate variability*” OR "heart rate interval" OR “aging” OR “RR variability” OR “cycle length variability” OR “heart period variability” OR “autonomic function” OR “vagal control” OR “lipid profile” OR “cholesterol” OR “diabetes” AND “mellitus” OR “blood glucose” OR “age” OR “obesity”) AND (EXCLUDE (DOCTYPE , "no") OR EXCLUDE (DOCTYPE , "cp") OR EXCLUDE (DOCTYPE , "ch") OR EXCLUDE (DOCTYPE , "bk"))

#3 AND (“muscular injury” OR {“musculoskeletal” AND “system”} OR “muscular pain” OR “chronic Pain” OR “acute pain” “acute injury” OR “muscular health”) AND (EXCLUDE (DOCTYPE , "no") OR EXCLUDE (DOCTYPE , "cp") OR EXCLUDE (DOCTYPE , "ch") OR EXCLUDE (DOCTYPE , "bk"))

#4 AND ({physical fitness} OR “exercise” OR “physical exertion” OR “muscular strength” OR “muscular endurance” OR “aerobic fitness*” OR “cardiorespiratory fitness*” OR “cardiorespiratory capacity*”) AND (EXCLUDE (DOCTYPE , "no") OR EXCLUDE (DOCTYPE , "cp") OR EXCLUDE (DOCTYPE , "ch") OR EXCLUDE (DOCTYPE , "bk"))

#5 AND ({work performance} OR “endurance*” OR “work fitness*” OR "work performance" OR "work classification" OR "occupational health" OR “employee health” OR “health, industrial” OR “industrial health” OR “occupational safety” OR “safety, occupational*”) AND (EXCLUDE (DOCTYPE , "no") OR EXCLUDE (DOCTYPE , "cp") OR EXCLUDE (DOCTYPE , "ch") OR EXCLUDE (DOCTYPE , "bk"))

#6 #1 AND #2 OR #1 AND #3 OR #1 AND #4 OR #1 AND #2 AND #3 OR #1 AND #2 AND #4 OR #1 AND #3 AND #4 OR #1 AND #2 AND #3 AND #4 OR #1 AND #5 OR #2 AND #5 OR # 3 AND #5 OR #3 AND #5

Web of sciences

#1 TOPIC:(Firefighter*)/ ("fire and rescue" OR firefighters OR fire fighter OR firefight OR firemen) a

#2 AND TOPIC:("cardiovascular" OR “cardiovascular abnormalities” OR “HRV*” OR “heart rate variability*” OR "heart rate interval" OR “aging” OR “RR variability” OR “cycle length variability” OR “heart period variability” OR “autonomic function” OR “vagal control” OR “lipid profile” OR “cholesterol” OR “diabetes” AND “mellitus” OR “blood glucose” OR “age” OR “obesity”)Refined by: [excluding] DOCUMENT TYPES: (PROCEEDINGS PAPER OR BOOK CHAPTER OR NOTE OR MEETING ABSTRACT)

#3 AND TOPIC:(“muscular injury” OR {“musculoskeletal” AND “system”} OR “muscular pain” OR “chronic Pain” OR “acute pain” “acute injury” OR “muscular

health”) Refined by: [excluding] DOCUMENT TYPES: (PROCEEDINGS PAPER OR BOOK CHAPTER OR NOTE OR MEETING ABSTRACT)

#4 AND TOPIC:("physical fitness" OR “exercise” OR “physical exertion” OR “muscular strength” OR “muscular endurance” OR “aerobic fitness” OR “cardiorespiratory fitness” OR “cardiorespiratory capacity”) Refined by: [excluding] DOCUMENT TYPES: (PROCEEDINGS PAPER OR BOOK CHAPTER OR NOTE OR MEETING ABSTRACT)

#5 AND TOPIC:(work performance OR “endurance*” OR “work fitness” OR "work performance" OR "work classification" OR "occupational health" OR “employee health” OR “health, industrial” OR “industrial health” OR “occupational safety” OR “safety, occupational*”) Refined by: [excluding] DOCUMENT TYPES: (PROCEEDINGS PAPER OR BOOK CHAPTER OR NOTE OR MEETING ABSTRACT)

#6 #1 AND #2 OR #1 AND #3 OR #1 AND #4 OR #1 AND #2 AND #3 OR #1 AND #2 AND #4 OR #1 AND #3 AND #4 OR #1 AND #2 AND #3 AND #4 OR #1 AND #5 OR #2 AND #5 OR # 3 AND #5 OR #3 AND #5

EBSCOHost

#1 Subject Terms:(“Firefighter” OR "fire and rescue" OR “firefighters” OR “fire fighter” OR “firefight” OR “firemen”) Field:(All text)

#2 AND ("cardiovascular” OR “cardiovascular abnormalities” OR “HRV*” OR “heart rate variability*” OR "heart rate interval" OR “aging” OR “RR variability” OR “cycle length variability” OR “heart period variability” OR “autonomic function” OR “vagal control” OR “lipid profile” OR “cholesterol” OR “diabetes” AND “mellitus” OR “blood glucose” OR “age” OR “obesity”) Field:(All text)

#3 AND (“muscular injury” OR {“musculoskeletal” AND “system”} OR “muscular pain” OR “chronic Pain” OR “acute pain” “acute injury” OR “muscular health”) Field (All text)

#4 AND (“physical fitness” OR “exercise” OR “physical exertion” OR “muscular strength” OR “muscular endurance” OR “aerobic fitness*” OR “cardiorespiratory fitness*” OR “cardiorespiratory capacity*”) Field:(All text)

#5 AND (work performance OR “endurance” OR “work fitness” OR "work performance" OR "work classification" OR "occupational health" OR “employee health” OR “health, industrial” OR “industrial health” OR “occupational safety” OR “safety, occupational”) Field:(All text)

#6 #1 AND #2 OR #1 AND #3 OR #1 AND #4 OR #1 AND #2 AND #3 OR #1 AND #2 AND #4 OR #1 AND #3 AND #4 OR #1 AND #2 AND #3 AND #4 OR #1 AND #5 OR #2 AND #5 OR # 3 AND #5 OR #3 AND #5

ScienceDirect

#1 Subject Terms:(“Firefighter” OR "fire and rescue" OR “firefighters” OR “fire fighter” OR “firefight” OR “firemen”) Field:(All text)

#2 AND ("cardiovascular” OR “cardiovascular abnormalities” OR “HRV*” OR “heart rate variability*” OR "heart rate interval" OR “aging” OR “RR variability” OR “cycle length variability” OR “heart period variability” OR “autonomic function” OR “vagal control” OR “lipid profile” OR “cholesterol” OR “diabetes” AND “mellitus” OR “blood glucose” OR “age” OR “obesity”) Field:(All text)

#3 AND (“muscular injury” OR {“musculoskeletal” AND “system”} OR “muscular pain” OR “chronic Pain” OR “acute pain” “acute injury” OR “muscular health”) Field (All text)

#4 AND (“physical fitness” OR “exercise” OR “physical exertion” OR “muscular strength” OR “muscular endurance” OR “aerobic fitness*” OR “cardiorespiratory fitness*” OR “cardiorespiratory capacity*”) Field:(All text)

#5 AND (work performance OR “endurance” OR “work fitness” OR "work performance" OR "work classification" OR "occupational health" OR “employee health” OR “health, industrial” OR “industrial health” OR “occupational safety” OR “safety, occupational”) Field:(All text)

#6 #1 AND #2 OR #1 AND #3 OR #1 AND #4 OR #1 AND #2 AND #3 OR #1 AND #2 AND #4 OR #1 AND #3 AND #4 OR #1 AND #2 AND #3 AND #4 OR #1 AND #5 OR #2 AND #5 OR # 3 AND #5 OR #3 AND #5
