# Supplementary material for: Effects of Cardiovascular Disease Risk Factors, Musculoskeletal Health, and Physical Fitness on Occupational Performance in Firefighters—A Systematic Review and Meta-Analysis
Source: Int J Environ Res Public Health. 2022 Sep 21;19(19):11946. doi: 10.3390/ijerph191911946 (PMC9564707; doi:10.3390/ijerph191911946)
Supplement: Supplementary file 1 [file ijerph-19-11946-s001.zip › Supplementary S2_eligbility screening.pdf]

Appendix 2

**General information**	
Title	
Author	
Study type	
Exposure assessed	
Outcome relevance	
Notes:	

**Study eligibility**			
Study characteristics	Eligibility criteria met		
	Yes	No	Unclear
Type of Study			
Type of participants			
Type of exposure			
Type of comparison			
Types of outcome measures			

INCLUDE	EXCLUDE
**Reason for exclusion:**	
