# Supplementary material for: Effects of Cardiovascular Disease Risk Factors, Musculoskeletal Health, and Physical Fitness on Occupational Performance in Firefighters—A Systematic Review and Meta-Analysis
Source: Int J Environ Res Public Health. 2022 Sep 21;19(19):11946. doi: 10.3390/ijerph191911946 (PMC9564707; doi:10.3390/ijerph191911946)
Supplement: Supplementary file 1 [file ijerph-19-11946-s001.zip › Supplementary S3_Data Extraction Sheets.pdf]

UNIVERSITY OF THE WESTERN CAPE

Private Bag X 17, Bellville 7535, South Africa

Tel: +27 21-959 2653, Fax: 27 21-959 3686,

E-mail: 3405618@myuwc.ac.za

DATA EXTRACTION SHEETS

General Description

Author	Target Population	Geographical Location	Aim	Problem Statement

Methodology

UNIVERSITY OF THE WESTERN CAPE

Private Bag X 17, Bellville 7535, South Africa

Tel: +27 21-959 2653, Fax: 27 21-959 3686,

E-mail: lleach@uwc.ac.za

Author	Study Design	Theoretical Underpinnings	Sampling Method and Size	Data Collection Methods and Instruments

UNIVERSITY OF THE WESTERN CAPE
Private Bag X 17, Bellville 7535, South Africa
Tel: +27 21-959 2653, Fax: 27 21-959 3686,
E-mail: lleach@uwc.ac.za

Findings and Analysis

Author	Methods of Data Analysis	Study Findings	Author's Conclusions
